# Supplementary material for: Leaps and bounds: geographical and ecological distance constrained the colonisation of the Afrotemperate by Erica
Source: BMC Evol Biol. 2019 Dec 5;19:222. doi: 10.1186/s12862-019-1545-6 (PMC6896773; doi:10.1186/s12862-019-1545-6)
Supplement: Supplementary file 8 — Additional file 8. Results of the different models under DEC + J and DEC (generally the better models compared to DIVA-like and BAYAREA-like-models). [file 12862_2019_1545_MOESM8_ESM.docx]

**Appendix 8:** Results of the different models: a) for the single best tree under DEC+J; b) for the single best tree under DEC; and c) for bootstrap trees under DEC+J. deltaAIC values are calculated overall across models for a given tree, and separately for the max areas/adjacency matrix, the biogeographic scenarios, and the distance models (“per comparison”). Models within deltaAIC=2 of the best score overall are bold underlined.

**a) Single best tree, DEC+J model.**

| **Model** | **Dispersal multiplier** | **LnL** | **d [1/Ma]** | **e [1/Ma]** | **j** | **AIC** | **deltaAIC overall** | **deltaAIC per comparison** |
| --- | --- | --- | --- | --- | --- | --- | --- | --- |
| no constraint |  | -69.98 | 0.0007 | 1E-12 | 0.0007 | 146 | 15 | 12.8 |
| Max area=2 |  | -69.9 | 0.0008 | 1E-12 | 0.0007 | 145.8 | 14.8 | 12.6 |
| Adjacency matrix |  | -63.61 | 0.0027 | 1E-12 | 0.0006 | 133.2 | 2.2 | 0 |
| Max area=2 + adjacency matrix |  | -63.6 | 0.0027 | 1E-12 | 0.0006 | 133.2 | 2.2 | 0 |
|  |  |  |  |  |  |  |  |  |
| **The following models are based on the best model above** | | | | | | | |  |
| **Stepping Stone (w=1)** | 0 | -96.18 | 0.008 | 1E-12 | 0.024 | 198.4 | 67.4 | 67.4 |
|  | 1 | -70.64 | 0.0054 | 1E-12 | 0.028 | 147.3 | 16.3 | 16.3 |
|  | 5 | -65.9 | 0.0059 | 1E-12 | 0.013 | 137.8 | 6.8 | 6.8 |
|  | 7.5 | -65.06 | 0.0061 | 1E-12 | 0.0085 | 136.1 | 5.1 | 5.1 |
|  | 10 | -64.52 | 0.0061 | 1E-12 | 0.0064 | 135 | 4 | 4 |
|  | **25** | **-63.34** | **0.0052** | **1E-12** | **0.0026** | **132.7** | **1.7** | **1.7** |
|  | **50** | **-63.14** | **0.004** | **1E-12** | **0.0013** | **132.3** | **1.3** | **1.3** |
|  |  |  |  |  |  |  |  |  |
| Cape to Cairo (w=1) | 0 | -83.07 | 0.0045 | 0.0006 | 0.0056 | 172.1 | 41.1 | 41.1 |
|  | 1 | -71.75 | 0.0035 | 1E-12 | 0.0032 | 149.5 | 18.5 | 18.5 |
|  | 5 | -67.49 | 0.0035 | 1E-12 | 0.0027 | 141 | 10 | 10 |
|  | 7.5 | -66.53 | 0.0035 | 1E-12 | 0.0025 | 139.1 | 8.1 | 8.1 |
|  | 10 | -65.89 | 0.0035 | 1E-12 | 0.0023 | 137.8 | 6.8 | 6.8 |
|  | 25 | -64.28 | 0.0034 | 1E-12 | 0.0017 | 134.6 | 3.6 | 3.6 |
|  | 50 | -63.63 | 0.0031 | 1E-12 | 0.0011 | 133.3 | 2.3 | 2.3 |
|  |  |  |  |  |  |  |  |  |
| **Drakensberg Melting-pot (w=1)** | 0 | -72.09 | 0.0033 | 0.0006 | 0.0037 | 150.2 | 19.2 | 19.2 |
|  | 1 | -64.09 | 0.0027 | 1E-12 | 0.0033 | 134.2 | 3.2 | 3.2 |
|  | **5** | **-62.52** | **0.0027** | **1E-12** | **0.0024** | **131** | **0** | **0** |
|  | **7.5** | **-62.62** | **0.0027** | **1E-12** | **0.0026** | **131.2** | **0.2** | **0.2** |
|  | **10** | **-62.52** | **0.0027** | **1E-12** | **0.0024** | **131** | **0** | **0** |
|  | **25** | **-62.51** | **0.0028** | **1E-12** | **0.0017** | **131** | **0** | **0** |

| **(*E. aborea* European)** | 25 | **-61.92** | **0.0024** | **1E-12** | **0.0021** | **129.8** | **x** | **x** |
| --- | --- | --- | --- | --- | --- | --- | --- | --- |
| **w =0.8** | **25** | **-62.61** | **0.0028** | **1E-12** | **0.0015** | **131.23** | **0.23** | **0.23** |
| **w =0.5** | **25** | **-62.88** | **0.0028** | **1E-12** | **0.0011** | **131.76** | **0.76** | **0.76** |
| **w =0.1** | **25** | **-63.43** | **0.0027** | **1E-12** | **0.00071** | **132.86** | **1.86** | **1.86** |
|  | **50** | **-62.88** | **0.0028** | **1E-12** | **0.0011** | **131.8** | **0.8** | **0.8** |
|  |  |  |  |  |  |  |  |  |
| **Geographic distance** | **As disp. probability (0 to 1)** | **-62.79** | **0.0029** | **1E-12** | **0.0012** | **131.58** | **0.58** | **0** |
|  | As distance (1 to x) | -72.37 | 0.0002 | 1E-12 | 0.00001 | 150.7 | 19.7 | 19.12 |
|  | As distance ^ -0.25 | -64.16 | 0.0034 | 1E-12 | 0.0013 | 134.3 | 3.3 | 2.72 |
|  | As distance ^ -1 | -71.40 | 0.004 | 1E-12 | 0.0032 | 148.8 | 17.8 | 17.22 |
|  | As distance ^ -2 | -83.18 | 0.0035 | 1E-12 | 0.0029 | 172.4 | 41.4 | 40.82 |
| Niche similarity | Schoener‘s D | -64.22 | 0.0047 | 1E-12 | 0.0013 | 134.4 | 3.4 | 2.82 |
| Niche plus distance as multipl. | As disp. probability/distance matrix | -64.01 | 0.0049 | 1E-12 | 0.0020 | 134.0 | 3.0 | 2.4 |

**b) Single best tree, DEC model.**

| Model | Dispersal multiplier | LnL | d [1/Ma] | e [1/Ma] | AIC | deltaAIC | deltaAIC per comparison |
| --- | --- | --- | --- | --- | --- | --- | --- |
| no constraint |  | -73.59 | 0.0011 | 1E-12 | 151.2 | 10 | 0.24 |
| Max area=2 |  | -73.48 | 0.0011 | 1E-12 | 150.96 | 9.76 | 0 |
| Adjacency matric |  | -76.72 | 0.0038 | 1E-12 | 157.44 | 16.24 | 6.48 |
| Max area=2 + adjacency matrix |  | -76.7 | 0.0039 | 1E-12 | 157.4 | 16.2 | 6.44 |
|  |  |  |  |  |  |  |  |
| **The following models are based on the best model above** | | | | | | |  |
| Stepping Stone (w=1) | 0 | -79.41 | 0.011 | 1E-12 | 162.8 | 21.6 | 21.6 |
|  | 1 | -76.63 | 0.01 | 1E-12 | 157.3 | 16.1 | 16.1 |
|  | 5 | -72.12 | 0.0076 | 1E-12 | 148.2 | 7 | 7 |
|  | 7.5 | -71.47 | 0.0066 | 1E-12 | 146.9 | 5.7 | 5.7 |
|  | 10 | -71.18 | 0.0058 | 1E-12 | 146.4 | 5.2 | 5.2 |
|  | 25 | -71.21 | 0.0034 | 1E-12 | 146.4 | 5.2 | 5.2 |
|  | 50 | -72.09 | 0.002 | 1E-12 | 148.2 | 7 | 7 |
|  |  |  |  |  |  |  |  |

| Cape to Cairo (w=1) | 0 | -89.65 | 0.0062 | 0.0005 | 183.3 | 42.1 | 42.1 |
| --- | --- | --- | --- | --- | --- | --- | --- |
|  | 1 | -76.7 | 0.0044 | 1E-12 | 157.4 | 16.2 | 16.2 |
|  | 5 | -73.14 | 0.0039 | 1E-12 | 150.3 | 9.1 | 9.1 |
|  | 7.5 | -72.43 | 0.0036 | 1E-12 | 148.9 | 7.7 | 7.7 |
|  | 10 | -72.03 | 0.0034 | 1E-12 | 148.1 | 6.9 | 6.9 |
|  | 25 | -71.52 | 0.0025 | 1E-12 | 147 | 5.8 | 5.8 |
|  | 50 | -72.09 | 0.0018 | 1E-12 | 148.2 | 7 | 7 |
|  |  |  |  |  |  |  |  |
| **Drakensberg Melting-pot (w=1)** | 0 | -77.02 | 0.004 | 0.0007 | 158 | 16.8 | 16.8 |
|  | 1 | -69.66 | 0.0035 | 1E-12 | 143.32 | 2.12 | 2.12 |
|  | **5** | **-68.66** | **0.0029** | **1E-12** | **141.3** | **0.1** | **0.1** |
|  | **7.5** | **-68.61** | **0.003** | **1E-12** | **141.2** | **0** | **0** |
| **(*E. aborea* not widespread)** | **7.5** | -68.54 | 0.003 | 1.00E-12 | 141.1 | x | x |
| **w =0.8** | **7.5** | **-68.77** | **0.0028** | **1E-12** | **141.5** | **0.3** | **0.3** |
| w =0.5 | 7.5 | -69.67 | 0.0022 | 1E-12 | 143.3 | 2.1 | 2.1 |
| w =0.1 | 7.5 | -72.48 | 0.0013 | 1E-12 | 149 | 7.8 | 7.8 |
|  | **10** | **-68.66** | **0.0029** | **1E-12** | **141.3** | **0.1** | **0.1** |
|  | **25** | **-69.52** | **0.0023** | **1E-12** | **143** | **1.8** | **1.8** |
|  | 50 | -72.09 | 0.0018 | 1E-12 | 148.2 | 7 | 7 |
|  |  |  |  |  |  |  |  |
| Geographic distance | As disp multiplier (0 to 1) | -69.68 | 0.0021 | 1E-12 | 143.4 | 2.2 | 0 |
|  | As Distance ( to x) | -90.3 | 0.000018 | 1E-12 | 184.6 | 43.4 | 41.2 |
| (Divalike * J was better) | As distance ^ -0.25 | -71.38 | 0.0023 | 1E-12 | 146.8 | 5.6 | 3.4 |
|  | As distance ^ -1 | -74.66 | 0.0054 | 1E-12 | 153.3 | 12.1 | 9.9 |
|  | As distance ^ -2 | -85.62 | 0.0052 | 1E-12 | 175.2 | 34 | 31.8 |
| Niche similarity | Schoener‘s D | -72.84 | 0.0024 | 1E-12 | 149.67 | 8.47 | 6.27 |
| Niche plus distance as multipl. | As disp. probability/distance matrix | -70.42 | 0.0038 | 1E-12 | 144.8 | 3.6 | 1.4 |

**c) Bootstrap trees, DEC+J model.**

| **BS-tree** | Dispersal multiplier | Biogeographic model | LnL | AIC | deltaAIC | deltaAIC per comp. |
| --- | --- | --- | --- | --- | --- | --- |
| **1_1** |  | No constraint | -69.53 | 145.1 | 15.7 | 10.2 |
|  |  | Max area=2 + adjacency matrix | -64.43 | 134.9 | 5.5 | 0 |
|  | 0 | Drakensberg melting pot | -69.39 | 144.8 | 15.4 | 13.3 |
|  |  | Cape to Cairo | -73.68 | 153.4 | 24 | 21.9 |
|  |  | Stepping stone | -94.05 | 194.1 | 64.7 | 62.6 |
|  | 0.01 | Drakensberg melting pot | -65.69 | 137.4 | 8 | 5.9 |
|  |  | Cape to Cairo | -68.86 | 143.7 | 14.3 | 12.2 |
|  |  | Stepping stone | -75.05 | 156.1 | 26.7 | 24.6 |
|  | 0.1 | Drakensberg melting pot | -64.05 | 134.1 | 4.7 | 2.6 |
|  |  | Cape to Cairo | -63.51 | 133 | 3.6 | **1.5** |
|  |  | Stepping stone | -68.7 | 143.4 | 14 | 11.9 |
|  | 0.25 | Drakensberg melting pot | -63.89 | 133.8 | 4.4 | 2.3 |
|  |  | Cape to Cairo | -62.74 | 131.5 | 2.1 | **0** |
|  |  | Stepping stone | -67.03 | 140.1 | 10.7 | 8.6 |
|  | 0.5 | Drakensberg melting pot | -64.04 | 134.1 | 4.7 | 2.6 |
|  |  | Cape to Cairo | -63.13 | 132.3 | 2.9 | **0.8** |
|  |  | Stepping stone | -65.63 | 137.3 | 7.9 | 5.8 |
|  | distance based | **Niche similarity** | **-62.62** | **131.2** | **1.8** | **1.8** |
|  |  | Pure distance | -62.75 | 131.5 | 2.1 | 2.1 |
|  |  | **Niche similarity + pure distance** | **-61.71** | **129.4** | **0** | **0** |
|  |  |  |  |  |  |  |
| **1_2** |  | No constraint | -64.62 | 135.2 | 17.3 | 11.3 |
|  |  | Max area=2 + adjacency matrix | -58.97 | 123.9 | 6 | 0 |
|  | 0 | Drakensberg melting pot | -64.93 | 135.9 | 18 | 16.9 |
|  |  | Cape to Cairo | -67.88 | 141.8 | 23.9 | 22.8 |
|  |  | Stepping stone | -93.66 | 193.3 | 75.4 | 74.3 |
|  | 0.01 | Drakensberg melting pot | -59.16 | 124.3 | 6.4 | 5.3 |
|  |  | Cape to Cairo | -61.87 | 129.7 | 11.8 | 10.7 |
|  |  | Stepping stone | -69.51 | 145 | 27.1 | 26 |
|  | 0.1 | Drakensberg melting pot | -57.65 | 121.3 | 3.4 | 2.3 |
|  |  | **Cape to Cairo** | **-56.87** | **119.7** | **1.8** | **0.7** |
|  |  | Stepping stone | -62.61 | 131.2 | 13.3 | 12.2 |
|  | 0.25 | Drakensberg melting pot | -57.73 | 121.5 | 3.6 | 2.5 |
|  |  | **Cape to Cairo** | **-56.52** | **119** | **1.1** | **0** |
|  |  | Stepping stone | -60.91 | 127.8 | 9.9 | 8.8 |
|  | 0.5 | Drakensberg melting pot | -58.2 | 122.4 | 4.5 | 3.4 |
|  |  | Cape to Cairo | -57.3 | 120.6 | 2.7 | 1.6 |
|  |  | Stepping stone | -59.85 | 125.7 | 7.8 | 6.7 |
|  | distance based | Niche similarity | -57.19 | 120.4 | 2.5 | 2.5 |
|  |  | **Pure distance** | **-56.85** | **119.7** | **1.8** | **1.8** |
|  |  | **Niche similarity + pure distance** | **-55.93** | **117.9** | **0** | **0** |
|  |  |  |  |  |  |  |
| **1_o** |  | No constraint | -68.72 | 143.4 | 19.9 | 11.4 |
|  |  | Max area=2 + adjacency matrix | -62.99 | 132 | 8.5 | 0 |
|  | 0 | Drakensberg melting pot | -76 | 158 | 34.5 | 34.5 |
|  |  | Cape to Cairo | -70.11 | 146.2 | 22.7 | 22.7 |
|  |  | Stepping stone | -99.88 | 205.8 | 82.3 | 82.3 |
|  | 0.01 | Drakensberg melting pot | -62.96 | 131.9 | 8.4 | 8.4 |
|  |  | Cape to Cairo | -62.14 | 130.3 | 6.8 | 6.8 |
|  |  | Stepping stone | -72.67 | 151.3 | 27.8 | 27.8 |
|  | 0.1 | Drakensberg melting pot | -61.51 | 129 | 5.5 | 5.5 |
|  |  | **Cape to Cairo** | **-58.73** | **123.5** | **0** | **0** |
|  |  | Stepping stone | -66.31 | 138.6 | 15.1 | 15.1 |
|  | 0.25 | Drakensberg melting pot | -61.65 | 129.3 | 5.8 | 5.8 |
|  |  | **Cape to Cairo** | **-59.45** | **124.9** | **1.4** | **1.4** |
|  |  | Stepping stone | -64.8 | 135.6 | 12.1 | 12.1 |
|  | 0.5 | Drakensberg melting pot | -62.17 | 130.3 | 6.8 | 6.8 |
|  |  | Cape to Cairo | -60.91 | 127.8 | 4.3 | 4.3 |
|  |  | Stepping stone | -63.85 | 133.7 | 10.2 | 10.2 |
|  | distance based | Niche similarity | -60.74 | 127.5 | 4 | 3.5 |
|  |  | Pure distance | -60.57 | 127.1 | 3.6 | 3.1 |
|  |  | **Niche similarity + pure distance** | **-59.02** | **124** | **0.5** | **0** |
|  |  |  |  |  |  |  |
| **2_1** |  | no constraint | -63.81 | 133.6 | 14.5 | 12.2 |
|  |  | no dispersal constraint | -57.69 | 121.4 | 2.3 | 0 |
|  | 0 | Drakensberg melting pot | -59.72 | 125.4 | 6.3 | 6.2 |
|  |  | Cape to Cairo | -66.2 | 138.4 | 19.3 | 19.2 |
|  |  | Stepping stone | -82.05 | 170.1 | 51 | 50.9 |
|  | 0.01 | Drakensberg melting pot | -58.06 | 122.1 | 3 | 2.9 |
|  |  | Cape to Cairo | -62.03 | 130.1 | 11 | 10.9 |
|  |  | Stepping stone | -67.05 | 140.1 | 21 | 20.9 |
|  | 0.1 | **Drakensberg melting pot** | **-56.59** | **119.2** | **0.1** | **0** |
|  |  | Cape to Cairo | -58.78 | 123.6 | 4.5 | 4.4 |
|  |  | Stepping stone | -59.95 | 125.9 | 6.8 | 6.7 |
|  | 0.25 | **Drakensberg melting pot** | **-56.61** | **119.2** | **0.1** | **0** |
|  |  | **Cape to Cairo** | **-57.5** | **121** | **1.9** | **1.8** |
|  |  | Stepping stone | -58.38 | 122.8 | 3.7 | 3.6 |
|  | 0.5 | **Drakensberg melting pot** | **-57** | **120** | **0.9** | **0.8** |
|  |  | **Cape to Cairo** | **-57.21** | **120.4** | **1.3** | **1.2** |
|  |  | Stepping stone | -57.74 | 121.5 | 2.4 | 2.3 |
|  | distance based | **Niche similarity** | **-57.57** | **121.1** | **2** | **2** |
|  |  | **Pure distance** | **-56.54** | **119.1** | **0** | **0** |
|  |  | **Niche similarity + pure distance** | **-57.18** | **120.4** | **1.3** | **1.3** |
|  |  |  |  |  |  |  |
| **2_2** |  | no constraint | x | 152.4 | 16.8 | 13.9 |
|  |  | no dispersal constraint | -66.27 | 138.5 | 2.9 | 0 |
|  | 0 | Drakensberg melting pot | -69.74 | 145.5 | 9.9 | 8.5 |
|  |  | Cape to Cairo | -72.49 | 151 | 15.4 | 14 |
|  |  | Stepping stone | -108.4 | 222.8 | 87.2 | 85.8 |
|  | 0.01 | Drakensberg melting pot | -67 | 140 | 4.4 | 3 |
|  |  | Cape to Cairo | -70.19 | 146.4 | 10.8 | 9.4 |
|  |  | Stepping stone | -74.48 | 155 | 19.4 | 18 |
|  | 0.1 | **Drakensberg melting pot** | **-65.54** | **137.1** | **1.5** | **0.1** |
|  |  | Cape to Cairo | -66.7 | 139.4 | 3.8 | 2.4 |
|  |  | Stepping stone | -68.75 | 143.5 | 7.9 | 6.5 |
|  | 0.25 | **Drakensberg melting pot** | **-65.51** | **137** | **1.4** | **0** |
|  |  | **Cape to Cairo** | **-65.57** | **137.1** | **1.5** | **0.1** |
|  |  | Stepping stone | -67.5 | 141 | 5.4 | 4 |
|  | 0.5 | **Drakensberg melting pot** | **-65.79** | **137.6** | **2** | **0.6** |
|  |  | **Cape to Cairo** | **-65.5** | **137** | **1.4** | **0** |
|  |  | Stepping stone | -66.66 | 139.3 | 3.7 | 2.3 |
|  | distance based | **Niche similarity** | **-65.75** | **137.5** | **1.9** | **1.9** |
|  |  | **Pure distance** | **-65.28** | **135.6** | **0** | **0** |
|  |  | **Niche similarity + pure distance** | **-65.18** | **136.4** | **0.8** | **0.8** |
|  |  |  |  |  |  |  |
| **2_o** |  | no constraint |  | 148.6 | 18 | 13.5 |
|  |  | no dispersal constraint | -64.57 | 135.1 | 4.5 | 0 |
|  | 0 | Drakensberg melting pot | -66.06 | 138.1 | 7.5 | 6.9 |
|  |  | Cape to Cairo | -66.43 | 138.9 | 8.3 | 7.7 |
|  |  | Stepping stone | -96.87 | 199.7 | 69.1 | 68.5 |
|  | 0.01 | Drakensberg melting pot | -64.84 | 135.7 | 5.1 | 4.5 |
|  |  | Cape to Cairo | -64.22 | 134.4 | 3.8 | 3.2 |
|  |  | Stepping stone | -74.9 | 155.8 | 25.2 | 24.6 |
|  | 0.1 | Drakensberg melting pot | -63.66 | 133.3 | 2.7 | 2.1 |
|  |  | **Cape to Cairo** | **-62.88** | **131.8** | **1.2** | 0.6 |
|  |  | Stepping stone | -68.47 | 142.9 | 12.3 | 11.7 |
|  | 0.25 | Drakensberg melting pot | -63.71 | 133.4 | 2.8 | 2.2 |
|  |  | **Cape to Cairo** | **-62.59** | **131.2** | **0.6** | 0 |
|  |  | Stepping stone | -66.85 | 139.7 | 9.1 | 8.5 |
|  | 0.5 | Drakensberg melting pot | -64.04 | 134.1 | 3.5 | 2.9 |
|  |  | **Cape to Cairo** | **-63.14** | **132.3** | **1.7** | 1.1 |
|  |  | Stepping stone | -65.61 | 137.2 | 6.6 | 6 |
|  | distance based | **Niche similarity** | **-63.18** | **132.4** | **1.8** | **1.8** |
|  |  | **Pure distance** | **-62.76** | **131.5** | **0.9** | **0.9** |
|  |  | **Niche similarity + pure distance** | **-62.28** | **130.6** | **0** | **0** |
|  |  |  |  |  |  |  |
| **o_1** |  | no constraint | -76.11 | 158.2 | 21.1 | 13.5 |
|  |  | no dispersal constraint | -69.33 | 144.7 | 7.6 | 0 |
|  | 0 | Drakensberg melting pot | -78.57 | 163.1 | 26 | 25.5 |
|  |  | Cape to Cairo | -78.26 | 162.5 | 25.4 | 24.9 |
|  |  | Stepping stone | -99.74 | 205.5 | 68.4 | 67.9 |
|  | 0.01 | Drakensberg melting pot | -69.74 | 145.5 | 8.4 | 7.9 |
|  |  | Cape to Cairo | -69.97 | 145.9 | 8.8 | 8.3 |
|  |  | Stepping stone | -77.47 | 160.9 | 23.8 | 23.3 |
|  | 0.1 | Drakensberg melting pot | -68.43 | 142.9 | 5.8 | 5.3 |
|  |  | **Cape to Cairo** | **-65.82** | **137.6** | **0.5** | 0 |
|  |  | Stepping stone | -72.32 | 150.6 | 13.5 | 13 |
|  | 0.25 | Drakensberg melting pot | -68.62 | 143.2 | 6.1 | 5.6 |
|  |  | **Cape to Cairo** | **-66.13** | **138.3** | **1.2** | **0.7** |
|  |  | Stepping stone | -71.46 | 148.9 | 11.8 | 11.3 |
|  | 0.5 | Drakensberg melting pot | -68.98 | 144 | 6.9 | 6.4 |
|  |  | Cape to Cairo | -67.39 | 140.8 | 3.7 | 3.2 |
|  |  | Stepping stone | -70.6 | 147.2 | 10.1 | 9.6 |
|  | distance based | Niche similarity | -66.94 | 139.9 | 2.8 | 2.8 |
|  |  | Pure distance | -67.19 | 140.4 | 3.3 | 3.3 |
|  |  | **Niche similarity + pure distance** | **-65.57** | **137.1** | **0** | **0** |
|  |  |  |  |  |  |  |
| **o_2** |  | no constraint | -68.75 | 158.2 | 31.9 | 25.6 |
|  |  | no dispersal constraint | -63.31 | 132.6 | 6.3 | 0 |
|  | 0 | Drakensberg melting pot | -69.45 | 144.9 | 18.6 | 18.2 |
|  |  | Cape to Cairo | -70.82 | 147.6 | 21.3 | 20.9 |
|  |  | Stepping stone | -101.2 | 208.4 | 82.1 | 81.7 |
|  | 0.01 | Drakensberg melting pot | -62.61 | 131.2 | 4.9 | 4.5 |
|  |  | Cape to Cairo | -65.03 | 136.1 | 9.8 | 9.4 |
|  |  | Stepping stone | -73.64 | 153.3 | 27 | 26.6 |
|  | 0.1 | Drakensberg melting pot | -61.29 | 128.6 | 2.3 | 1.9 |
|  |  | **Cape to Cairo** | **-60.44** | **126.9** | **0.6** | **0.2** |
|  |  | Stepping stone | -66.54 | 139.1 | 12.8 | 12.4 |
|  | 0.25 | Drakensberg melting pot | -61.58 | 129.2 | 2.9 | 2.5 |
|  |  | **Cape to Cairo** | **-60.37** | **126.7** | **0.4** | **0** |
|  |  | Stepping stone | -64.91 | 135.8 | 9.5 | 9.1 |
|  | 0.5 | Drakensberg melting pot | -62.26 | 130.5 | 4.2 | 3.8 |
|  |  | Cape to Cairo | -61.4 | 128.8 | 2.5 | 2.1 |
|  |  | Stepping stone | -64 | 134 | 7.7 | 7.3 |
|  | distance based | Niche similarity | -61.61 | 129.2 | 2.9 | 2.9 |
|  |  | **Pure distance** | **-60.96** | **127.9** | **1.6** | **1.6** |
|  |  | **Niche similarity + pure distance** | **-60.17** | **126.3** | **0** | **0** |
|  |  |  |  |  |  |  |
| **o_o** |  | no constraint | -71.04 | 148.1 | 19.7 | 13.7 |
|  |  | no dispersal constraint | -64.2 | 134.4 | 6 | 0 |
|  | 0 | Drakensberg melting pot | -73.69 | 153.4 | 25 | 24 |
|  |  | Cape to Cairo | -72.9 | 151.8 | 23.4 | 22.4 |
|  |  | Stepping stone | -92.02 | 190 | 61.6 | 60.6 |
|  | 0.01 | Drakensberg melting pot | -64.96 | 135.9 | 7.5 | 6.5 |
|  |  | Cape to Cairo | -66.98 | 140 | 11.6 | 10.6 |
|  |  | Stepping stone | -73.31 | 152.6 | 24.2 | 23.2 |
|  | 0.1 | Drakensberg melting pot | -63.45 | 132.9 | 4.5 | 3.5 |
|  |  | **Cape to Cairo** | **-62.06** | **130.1** | **1.7** | **0.7** |
|  |  | Stepping stone | -67.8 | 141.6 | 13.2 | 12.2 |
|  | 0.25 | Drakensberg melting pot | -63.43 | 132.9 | 4.5 | 3.5 |
|  |  | **Cape to Cairo** | **-61.72** | **129.4** | **1** | **0** |
|  |  | Stepping stone | -66.57 | 139.1 | 10.7 | 9.7 |
|  | 0.5 | Drakensberg melting pot | -63.71 | 133.4 | 5 | 4 |
|  |  | Cape to Cairo | -62.51 | 131 | 2.6 | 1.6 |
|  |  | Stepping stone | -65.38 | 136.8 | 8.4 | 7.4 |
|  | distance based | Niche similarity | -62.33 | 130.7 | 2.3 | 2.3 |
|  |  | **Pure distance** | **-62.18** | **130.4** | **2** | **2** |
|  |  | **Niche similarity + pure distance** | **-61.18** | **128.4** | **0** | **0** |

**d) Single best tree - rarefaction, DEC+J model.**

| Pruned tree 1 - deleted tips:  carnea_ATsn5, scoparia_AH, spiculifolia_AS57234, umbellata_DS, ciliaris_c_ANA, terminalis_a_ANA, cinerea_a_ANA, multiflora_a_ANA, australis_b_ANA, hexandra_RC465, pleiotricha_RC461, filago_BG93, occulta_EO, plumigera_EO11341, lithophila_MP1301, brachysepala_EO12727, doliiformis_MP797, intermedia_MM5082, discolor_heb_MP1214, astroites_EO12758, dolfiana_MP1297, walkeri_MP1237, transparens_MP893, bicolor_MP1098, stagnalis_sta_MP668, nudiflora_MP802, axillaris_MP1052, adunca_EO12746, cruenta_MP745, collina_EO12613, recurvifolia_EO12475a, sp_nov_MP1291, floccifera_MP987, copiosa_BG610, lavandulifolia_EO12506, colorans_EO12717, inordinata_EO11823, fimbriata_MP606, nevillei_MP1056, palliiflora_EO12533, uberiflora_BG586, abietina_abi_MP1013, bruniifolia_EO12460, parviporandra_MP877, areolata_EO12502, brevifolia_EO12459, physophylla_EO11418, exleeana_EO12499, obtusata_EO12458, versicolor_ver_MP1232, pulchella_MP736, tomentosa_MP961, chrysocodon_ANA, strigilifolia_MP619, savileae_MP975, hirtiflora_MP958, totta_MP525, pogonanthera_EO12835, patersonii_a_ANA, acuta_MP506, corifolia_ANA, lanata_MP1220, eremioides_MP533, cameronii_ANA, petrophila_EO7592, daphniflora_MP567, quadrisulcata_MP1031, atrovinosa_MP864, brachialis_MP734, curtophylla_EO12750, leptopus_ANA, hanekomii_EO11172, innovans_MP918, parviflora_EO12492, baueri_bau_MP1233, grata_MP879, regia_reg_MP922, mira_MP1257, caprina_EO12772, eustacei_MP1259, holosericea_EO12842, verticillata_ANA, paniculata_MP1274, patens_EO12457, autumnalis_MP665, duthieae_ANA, chionophila_MP790, curviflora_MP765, amoena_MP1032, adnata_MP501, lehmannii_MP625, oxycoccifolia_MP1275, coccinea_coc_MP598, hispiduloides_EO11544, ecklonii_EO12739, fastigiata_MP830, garciae_MP1253, florifera_EO12536, trichophora_EO12701, globiceps_con_EO12519, ocellata_MP574, gnaphaloides_MP511, erasmia_MP874, sparsa_BG602, ventricosa_MP713, paucifolia_cil_EO12528, scytophylla_MP1021, malmesburiensis_EO12575, elimensis_EO12843, coacervata_MP761, pannosa_EO12490, leucanthera_EO12452, haematocodon_MP1033, oliveri_MP1278, flacca_MP840, velatiflora_EO12547, rugata_EO12516, passerinae_MP1302, cumuliflora_EO12699, fontana_MP1069, calycina_EO12532, haemastoma_MP871, ovina_EO12487, lateralis_MP721, accommodata_EO11382, pinea_MP789, deflexa_MP1247, zebrensis_EO12787, phillipsii_MP794, montis_hominis_EO11827, eriophoros_EO12478, lachnaeifolia_MP994, lateriflora_EO12482, parilis_MP751, russakiana_MP684, bokkeveldia_EO12769, leucopelta_EO12598, revoluta_BT13679, evansii_MP641, frigida_MP658, rivularis_BB13936, glaphyra_MP647, alopecurus_MP630, algida_MP645, hillburtii_EO12593 | | | | | | | | |
| --- | --- | --- | --- | --- | --- | --- | --- | --- |
| **Model** | **Dispersal multiplier** | **LnL** | **d [1/Ma]** | **e [1/Ma]** | **j** | **AIC** | **deltaAIC overall** | **deltaAIC per comparison** |
| no constraint |  | -63,29 | 0,0008 | 1,00E-12 | 0,0015 | 132,7 | 15 | 10,2 |
| Max area=2 |  | -63,2 | 8,00E-04 | 1,00E-12 | 0,0015 | 132,5 | 14,8 | 10 |
| Adjacency matrix |  | -58,2 | 2,90E-03 | 1,00E-12 | 0,0013 | 122,5 | 4,8 | 0 |
| Max area=2 + adjacency matrix |  | -58,19 | 2,90E-03 | 1,00E-12 | 0,0013 | 122,5 | 4,8 | 0 |
|  |  |  |  |  |  |  |  |  |
| **The following models are based on the best model above** | | | | | | | | |
| Stepping Stone (w=1) | 0 | -86,77 | 7,10E-03 | 1,00E-12 | 0,16 | 179,6 | 61,9 | 61,9 |
|  | 1 | -63,47 | 6,60E-03 | 1,00E-12 | 0,042 | 133 | 15,3 | 15,3 |
|  | 5 | -58,87 | 6,50E-03 | 1,00E-12 | 0,022 | 123,8 | 6,1 | 6,1 |
|  | 7,5 | -58,2 | 6,40E-03 | 1,00E-12 | 0,016 | 122,5 | 4,8 | 4,8 |
|  | 10 | -57,84 | 6,40E-03 | 1,00E-12 | 0,013 | 121,8 | 4,1 | 4,1 |
|  | 25 | -57,26 | 5,50E-03 | 1,00E-12 | 0,0053 | 120,6 | 2,9 | 2,9 |
|  | 50 | -57,45 | 0,0043 | 1,0e-12 | 0,0026 | 121 | 3,3 | 3,3 |
|  |  |  |  |  |  |  |  |  |
| Cape to Cairo (w=1) | 0 | -74,9 | 0,0048 | 0,0008 | 0,0098 | 155,9 | 38,2 | 38,2 |
|  | 1 | -64,81 | 3,30E-03 | 1,00E-12 | 0,0064 | 135,7 | 18 | 18 |
|  | 5 | -60,97 | 3,50E-03 | 1,00E-12 | 0,0055 | 128 | 10,3 | 10,3 |
|  | 7,5 | -60,09 | 3,60E-03 | 1,00E-12 | 0,0051 | 126,3 | 8,6 | 8,6 |
|  | 10 | -59,54 | 3,60E-03 | 1,00E-12 | 0,0047 | 125,1 | 7,4 | 7,4 |
|  | 25 | -58,23 | 3,50E-03 | 1,00E-12 | 0,0034 | 122,5 | 4,8 | 4,8 |
|  | 50 | -57,89 | 3,30E-03 | 1,00E-12 | 0,0022 | 121,8 | 4,1 | 4,1 |
|  |  |  |  |  |  |  |  |  |
| **Drakensberg Melting-pot (w=1)** | 0 | -64,64 | 0,0035 | 0,0008 | 0,0073 | 135,4 | 17,7 | 17,7 |
|  | 1 | -57,08 | 2,70E-03 | 1,00E-12 | 0,0067 | 120,2 | 2,5 | 2,5 |
|  | **5** | **-55,83** | **2,80E-03** | **1,00E-12** | **0,005** | **117,7** | **0** | **0** |
|  | **7,5** | **-55,85** | **2,70E-03** | **1,00E-12** | **0,0054** | **117,8** | **0,1** | **0,1** |
|  | **10** | **-55,83** | **2,80E-03** | **1,00E-12** | **0,005** | **117,7** | **0** | **0** |
|  | **25** | **-56,19** | **2,80E-03** | **1,00E-12** | **0,0035** | **118,5** | **0,8** | **0,8** |
| (E, aborea European) | 25 |  |  |  |  |  |  |  |
| w =0,8 | 25 |  |  |  |  |  |  |  |
| w =0,5 | 25 |  |  |  |  |  |  |  |
| w =0,1 | 25 |  |  |  |  |  |  |  |
|  | 50 | -56,98 | 0,0029 | 1,00E-12 | 0,0023 | 120 | 2,3 | 2,3 |
|  |  |  |  |  |  |  |  |  |
| **Geographic distance** | **As disp, probability (0 to 1)** | -59,87 | 0,0042 | 1,00E-12 | 0,0022 | 125,8 | 8,1 | 3,4 |
|  | As distance (1 to x) | -67,87 | 3,00E-04 | 1,00E-12 | 1,50E-05 | 141,8 | 24,1 | 19,4 |
|  | As distance ^ -0,25 | -58,34 | 3,60E-03 | 1,00E-12 | 0,0027 | 122,8 | 5,1 | 0,4 |
|  | As distance ^ -1 | -65,02 | 0,0042 | 1,00E-12 | 0,0059 | 136,1 | 18,4 | 13,7 |
|  | As distance ^ -2 | -76,04 | 3,50E-03 | 1,00E-12 | 0,0057 | 158,1 | 40,4 | 35,7 |
| Niche similarity | Schoener‘s D | -58,69 | 5,00E-03 | 1,00E-12 | 0,0026 | 123,4 | 5,7 | 1 |
| Niche plus distance as multipl, | As disp, probability/distance matrix | -58,17 | 5,10E-03 | 1,00E-12 | 0,004 | 122,4 | 4,7 | 0 |

| Pruned tree 2 - deleted tips:  maderensis_AH, ciliaris_c_ANA, vagans_MP972, manipuliflora_a_ANA, cinerea_a_ANA, tetralix_c_ANA, erigena_a_ANA, umbellata_DS, australis_b_ANA, whyteana_A4, hexandra_RC465, johnstoniana_RC464, trichophora_EO12701, verticillata_ANA, cylindrica_MP1240, recurvifolia_EO12475a, oresigena_MP759, pubescens_EO12503, spectabilis_MP929, coacervata_MP761, perplexa_EO12788, pogonanthera_EO12835, bokkeveldia_EO12769, paniculata_MP1274, vernicosa_MP928, rhodopis_BAB13, remota_EO10386, dodii_EO11417, leucosiphon_MP776, ustulescens_RT1553, karwyderi_EO12718, brevifolia_EO12459, riparia_MP908, chionodes_EO11699, copiosa_BG610, ferrea_EO12494, trichroma_EO12517, ovina_EO12487, desmantha_MP562, scytophylla_MP1021, aneimena_EO12757, ampullacea_MP1277, sessiliflora_MP604, plumigera_EO11341, krugeri_EO12807, albescens_MP898, dianthifolia_MP583, lanata_MP1220, greyi_EO12501, densifolia_BG591, heleogena_MP1064, pageana_ANA, sitiens_MP827, glandulipila_MP521, bodkinii_TdV204, caprina_EO12772, tomentosa_MP961, spumosa_MP978, polycoma_FR, thimifolia_CM7, tumida_MP755, capensis_MP1047, doliiformis_MP797, glutinosa_MP687, nutans_BG599, ardens_MP1076, agglutinans_EO7679, pyxidiflora_ANA, cereris_MP863, nematophylla_EO12747, blandfordii_MM4208, subdivaricata_MP671, banksii_com_ANA, stagnalis_sta_MP668, halicacaba_ANA, barbigeroides_MP735, nana_ANA, fontana_MP1069, discolor_heb_MP1214, capillaris_MP1066, petiolaris_EO12783, viscaria_vis_MdV4, capitata_ANA, villosa_EO11394, macowanii_mac_MP810, phillipsii_MP794, multumbellifera_MP822, russakiana_MP684, umbratica_EO12760, baueri_bau_MP1233, bolusiae_ANA, ventricosa_MP713, rugata_EO12516, vallis_fluminis_EO12761, bruniifolia_EO12460, lasciva_MP906, laeta_MP1045, astroites_EO12758, subcapitata_MP1042, garciae_MP1253, leptopus_ANA, atrovinosa_MP864, plukenetii_plu_EO, turgida_S1962, hansfordii_MP1239, inordinata_EO11823, vestita_EO12702, amidae_EO12272, arachnocalyx_EO12453, pubigera_MP572, autumnalis_MP665, rusticula_EO12471, deflexa_MP1247, wendlandiana_EO12731, acuta_MP506, argentea_EO12475, orientalis_EO12608, curviflora_MP765, zeyheriana_EO, humidicola_EO11353, quadrangularis_MP620, bolusanthus_DEB1720, adnata_MP501, magnisylvae_EO10708, occulta_EO, lignosa_EO11763, denticulata_MP799, filamentosa_EO12728, prolata_EO12748, paucifolia_cil_EO12528, holosericea_EO12842, clavisepala_ANA, cameronii_ANA, perspicua_per_MP821, gerhardii_EO12700, chartacea_EO11408, caffrorum_MP644, alopecurus_MP630, algida_MP645, evansii_MP641, subverticillaris_EO12625, holtii_TO, psittacina_IJ1237, leucopelta_EO12598, woodii_DB1444 | | | | | | | | |
| --- | --- | --- | --- | --- | --- | --- | --- | --- |
| **Model** | **Dispersal multiplier** | **LnL** | **d [1/Ma]** | **e [1/Ma]** | **j** | **AIC** | **deltaAIC overall** | **deltaAIC per comparison** |
| no constraint |  | -58,63 | 0,0006 | 1,00E-12 | 0,0017 | 123,3 | 13,7 | 8,8 |
| Max area=2 |  | -58,53 | 6,00E-04 | 1,00E-12 | 0,0017 | 123,1 | 13,5 | 8,6 |
| Adjacency matrix |  | -54,22 | 2,60E-03 | 1,00E-12 | 0,0013 | 114,5 | 4,9 | 0 |
| Max area=2 + adjacency matrix |  | -54,21 | 2,60E-03 | 1,00E-12 | 0,0013 | 114,5 | 4,9 | 0 |
|  |  |  |  |  |  |  |  |  |
| **The following models are based on the best model above** | | | | | | | | |
| Stepping Stone (w=1) | 0 | -83,59 | 1,10E+01 | 1,00E-12 | 0,040 | 173,3 | 63,7 | 63,7 |
|  | 1 | -59,14 | 7,70E-03 | 1,00E-12 | 0,030 | 124,4 | 14,8 | 14,8 |
|  | 5 | -54,61 | 6,80E-03 | 1,00E-12 | 0,017 | 115,3 | 5,7 | 5,7 |
|  | 7,5 | -53,9 | 6,50E-03 | 1,00E-12 | 0,013 | 113,9 | 4,3 | 4,3 |
|  | 10 | -53,53 | 6,20E-03 | 1,00E-12 | 0,011 | 113,1 | 3,5 | 3,5 |
|  | 25 | -53,03 | 5,10E-03 | 1,00E-12 | 0,005 | 112,1 | 2,5 | 2,5 |
|  | 50 | -53,35 | 3,90E-03 | 1,00E-12 | 0,0026 | 112,8 | 3,2 | 3,2 |
|  |  |  |  |  |  |  |  |  |
| Cape to Cairo (w=1) | 0 | -69,84 | 0,0045 | 0,0008 | 0,0089 | 145,8 | 36,2 | 36,2 |
|  | 1 | -59,68 | 2,40E-03 | 1,00E-12 | 0,0069 | 125,4 | 15,8 | 15,8 |
|  | 5 | -56,42 | 2,70E-03 | 1,00E-12 | 0,0061 | 118,9 | 9,3 | 9,3 |
|  | 7,5 | -55,68 | 2,70E-03 | 1,00E-12 | 0,0057 | 117,4 | 7,8 | 7,8 |
|  | 10 | -55,21 | 2,80E-03 | 1,00E-12 | 0,0053 | 116,5 | 6,9 | 6,9 |
|  | 25 | -54,14 | 2,80E-03 | 1,00E-12 | 0,0037 | 114,4 | 4,8 | 4,8 |
|  | 50 | -53,93 | 2,80E-03 | 1,00E-12 | 0,0024 | 113,9 | 4,3 | 4,3 |
|  |  |  |  |  |  |  |  |  |
| **Drakensberg Melting-pot (w=1)** | 0 | -60,86 | 0,0031 | 0,0008 | 0,0072 | 127,8 | 18,2 | 18,2 |
|  | 1 | -53,01 | 2,30E-03 | 1,00E-12 | 0,0065 | 112,1 | 2,5 | 2,5 |
|  | **5** | **-51,75** | **2,30E-03** | **1,00E-12** | **0,0052** | **109,6** | **0** | **0** |
|  | **7,5** | **-51,77** | **2,30E-03** | **1,00E-12** | **0,0055** | **109,6** | **0** | **0** |
|  | **10** | **-51,75** | **2,30E-03** | **1,00E-12** | **0,0052** | **109,6** | **0** | **0** |
|  | **25** | **-52,16** | **2,40E-03** | **1,00E-12** | **0,0037** | **110,4** | **0,8** | **0,8** |
| (E, aborea European) | 25 |  |  |  |  |  |  |  |
| w =0,8 | 25 |  |  |  |  |  |  |  |
| w =0,5 | 25 |  |  |  |  |  |  |  |
| w =0,1 | 25 |  |  |  |  |  |  |  |
|  | 50 | -53,01 | 0,0025 | 1,00E-12 | 0,0024 | 112,1 | 2,5 | 2,5 |
|  |  |  |  |  |  |  |  |  |
| Geographic distance | As disp, probability (0 to 1) | -55,53 | 3,30E-03 | 1,00E-12 | 0,0023 | 117,1 | 7,5 | 2,9 |
|  | As distance (1 to x) | -63,25 | 3,00E-04 | 1,00E-12 | 1,40E-05 | 132,6 | 23 | 18,4 |
|  | As distance ^ -0,25 | -54,21 | 3,10E-03 | 1,00E-12 | 0,0028 | 114,5 | 4,9 | 0,3 |
|  | As distance ^ -1 | -59,81 | 0,0033 | 1,00E-12 | 0,0062 | 125,7 | 16,1 | 11,5 |
|  | As distance ^ -2 | -69,05 | 3,30E-03 | 1,00E-12 | 0,0053 | 144,2 | 34,6 | 30 |
| Niche similarity | Schoener‘s D | -54,65 | 4,40E-03 | 1,00E-12 | 0,0026 | 115,4 | 5,8 | 1,2 |
| Niche plus distance as multipl, | As disp, probability/distance matrix | -54,08 | 4,30E-03 | 1,00E-12 | 0,0042 | 114,2 | 4,6 | 0 |

| Pruned tree 3 - deleted tips:  manipuliflora_a_ANA, erigena_a_ANA, australis_b_ANA, vagans_MP972, ciliaris_c_ANA, multiflora_a_ANA, carnea_ATsn5, umbellata_DS, cinerea_a_ANA, trimera_MsnA, simii_RC466, microdonta_A5, adunca_EO12746, nudiflora_MP802, flacca_MP840, praecox_MP795, spumosa_MP978, bracteolaris_MP577, collina_EO12613, magistrati_EO11750, brevifolia_EO12459, abietina_abi_MP1013, artemisioides_MP551, acuta_MP506, strigosa_MP673, leonis_RTsn, turgida_S1962, caprina_EO12772, melastoma_mel_MP773, ventricosa_MP713, leucodesmia_MP724, magnisylvae_EO10708, triflora_MP564, interrupta_MP911, penduliflora_MP923, palliiflora_EO12533, parviporandra_MP877, sparrmanii_ANA, atrovinosa_MP864, sessiliflora_MP604, scytophylla_MP1021, daphniflora_MP567, recurvifolia_EO12475a, vallis_fluminis_EO12761, fascicularis_fac_MP809, cyrilliflora_CM10, simulans_ANA, dolfiana_MP1297, empetrina_EO12786, diaphana_BG611, modesta_ANA, gibbosa_ANA, oxycoccifolia_MP1275, florifera_EO12536, zeyheriana_EO, arcuata_MP523, leucosiphon_MP776, duthieae_ANA, rosacea_gla_BG628, stokoeanthus_EO4790, copiosa_BG610, annectens_ANA, corydalis_MP1005, recta_ANA, garciae_MP1253, trichophylla_EO10906, pogonanthera_EO12835, retorta_ANA, cumuliflora_EO12699, cyathiformis_a_ANA, maderi_MP757, hibbertii_MP982, coarctata_MP590, riparia_MP908, exleeana_EO12499, alfredii_FR, subulata_MdV2, bolusanthus_DEB1720, regerminans_MP576, albertyniae_MP927, ampullacea_MP1277, intermedia_MM5082, wendlandiana_EO12731, remota_EO10386, stagnalis_sta_MP668, humifusa_MP846, pulvinata_MP1304, goatcheriana_dra_EO12694, sonderiana_MP756, zebrensis_EO12787, shannonii_TdV262, angulosa_S2105, alexandri_EO12449, prolata_EO12748, bokkeveldia_EO12769, russakiana_MP684, leptopus_ANA, fausta_MP663, lucida_MP690, hottentotica_EO, monsoniana_MP986, cereris_MP863, deflexa_MP1247, tradouwensis_MP903, plumigera_EO11341, fontana_MP1069, lignosa_EO11763, accommodata_EO11382, curvifolia_MP700, rigidula_MP534, patersonii_a_ANA, ardens_MP1076, barbigeroides_MP735, mammosaP_MP951, ustulescens_RT1553, peltata_MP1231, bruniades_EO12465, gerhardii_EO12700, squarrosa_EO11742, pannosa_EO12490, chrysocodon_ANA, massonii_MP811, lasciva_MP906, pycnantha_MP1011, setacea_MP589, propendens_EO12464, cruenta_MP745, nubigena_MP868, paucifolia_cil_EO12528, gracilipes_MM5014, atromontana_EO12544, lepidota_MP541, ovina_EO12487, glabella_la_EO11224, physodes_ANA, velatiflora_EO12547, frigida_MP658, dominans_MP648, drakensbergensis_DB1443, woodii_DB1444, tysonii_EO12583, atherstonei_EO12261, glaphyra_MP647, cooperi_EO12588, oatesii_ANA | | | | | | | | |
| --- | --- | --- | --- | --- | --- | --- | --- | --- |
| **Model** | **Dispersal multiplier** | **LnL** | **d [1/Ma]** | **e [1/Ma]** | **j** | **AIC** | **deltaAIC overall** | **deltaAIC per comparison** |
| no constraint |  | -62,36 | 0,0008 | 1,00E-12 | 0,0015 | 130,8 | 15,3 | 10,1 |
| Max area=2 |  | -62,31 | 8,00E-04 | 1,00E-12 | 0,0015 | 130,7 | 15,2 | 10,0 |
| Adjacency matrix |  | -57,3 | 2,90E-03 | 1,00E-12 | 0,0013 | 120,7 | 5,2 | 0 |
| Max area=2 + adjacency matrix |  | -57,29 | 2,90E-03 | 1,00E-12 | 0,0013 | 120,7 | 5,2 | 0 |
|  |  |  |  |  |  |  |  |  |
| **The following models are based on the best model above** | | | | | | | | |
| Stepping Stone (w=1) | 0 | -85,17 | 1,10E+01 | 1,00E-12 | 0,050 | 176,4 | 60,9 | 60,9 |
|  | 1 | -62,27 | 7,10E-03 | 1,00E-12 | 0,040 | 130,6 | 15,1 | 15,1 |
|  | 5 | -57,81 | 6,80E-03 | 1,00E-12 | 0,021 | 121,7 | 6,2 | 6,2 |
|  | 7,5 | -57,16 | 6,70E-03 | 1,00E-12 | 0,016 | 120,4 | 4, | 4,9 |
|  | 10 | -56,81 | 6,60E-03 | 1,00E-12 | 0,013 | 119,7 | 4,2 | 4,2 |
|  | 25 | -56,28 | 5,60E-03 | 1,00E-12 | 0,0054 | 118,6 | 3,1 | 3,1 |
|  | 50 | -56,51 | 4,30E-03 | 1,00E-12 | 0,0027 | 119,1 | 3,6 | 3,6 |
|  |  |  |  |  |  |  |  |  |
| Cape to Cairo (w=1) | 0 | -61,33 | 0,0027 | 1,00E-12 | 0,01 | 128,7 | 13,2 | 13,2 |
|  | 1 | -58,82 | 3,10E-03 | 1,00E-12 | 0,0095 | 123,7 | 8,2 | 8,2 |
|  | 5 | -57,93 | 3,10E-03 | 1,00E-12 | 0,0082 | 121,9 | 6,4 | 6,4 |
|  | 7,5 | -57,83 | 3,20E-03 | 1,00E-12 | 0,0073 | 121,7 | 6,2 | 6,2 |
|  | 10 | -57,75 | 3,20E-03 | 1,00E-12 | 0,0064 | 121,6 | 6,1 | 6,1 |
|  | 25 | -57,19 | 3,50E-03 | 1,00E-12 | 0,0036 | 120,4 | 4,9 | 4,9 |
|  | 50 | -56,94 | 3,30E-03 | 1,00E-12 | 0,0023 | 120 | 4,5 | 4,5 |
|  |  |  |  |  |  |  |  |  |
| **Drakensberg Melting-pot (w=1)** | **0** | **-55,23** | **0,0023** | **1,00E-12** | **0,0094** | **116,5** | **1** | **1** |
|  | **1** | **-55,05** | **2,40E-03** | **1,00E-12** | **0,0085** | **116,2** | **0,7** | **0,7** |
|  | **5** | **-54,76** | **2,70E-03** | **1,00E-12** | **0,0053** | **115,6** | **0,1** | **0,1** |
|  | **7,5** | **-54,73** | **2,70E-03** | **1,00E-12** | **0,0058** | **115,5** | **0** | **0** |
|  | **10** | **-54,76** | **2,70E-03** | **1,00E-12** | **0,0053** | **115,6** | **0,1** | **0** |
|  | **25** | **-55,22** | **2,90E-03** | **1,00E-12** | **0,0036** | **116,5** | **1** | **0,9** |
| (E, aborea European) | 25 |  |  |  |  |  |  |  |
| w =0,8 | 25 |  |  |  |  |  |  |  |
| w =0,5 | 25 |  |  |  |  |  |  |  |
| w =0,1 | 25 |  |  |  |  |  |  |  |
|  | 50 | -56,04 | 0,0029 | 1,00E-12 | 0,0023 | 118,2 | 2,7 | 2,6 |
|  |  |  |  |  |  |  |  |  |
| **Geographic distance** | **As disp, probability (0 to 1)** | -58,96 | 0,0042 | 1,00E-12 | 0,0022 | 124 | 8,5 | 3,4 |
|  | As distance (1 to x) | -67,06 | 3,00E-04 | 6,90E-11 | 1,60E-05 | 140,2 | 24,7 | 19,6 |
|  | As distance ^ -0,25 | -57,45 | 3,50E-03 | 1,00E-12 | 0,0027 | 121 | 5,5 | 0,4 |
|  | As distance ^ -1 | -64,05 | 0,0041 | 1,00E-12 | 0,006 | 134,2 | 18,7 | 13,6 |
|  | As distance ^ -2 | -73,63 | 3,40E-03 | 1,00E-12 | 0,0057 | 153,3 | 37,8 | 32,7 |
| Niche similarity | Schoener‘s D | -57,81 | 4,90E-03 | 1,00E-12 | 0,0027 | 121,7 | 6,2 | 1,1 |
| Niche plus distance as multipl, | As disp, probability/distance matrix | -57,29 | 5,00E-03 | 1,00E-12 | 0,0041 | 120,6 | 5,1 | 0 |

| Pruned tree 4 - deleted tips:  spiculifolia_AS57234, umbellata_DS, australis_b_ANA, manipuliflora_a_ANA, scoparia_AH, mackayana_b_ANA, carnea_ATsn5, terminalis_a_ANA, multiflora_a_ANA, pleiotricha_RC461, lanceolifera_RC463, silvatica_A2, walkeri_MP1237, nevillei_MP1056, tumida_MP755, accommodata_EO11382, flacca_MP840, maderi_MP757, pogonanthera_EO12835, shannonii_TdV262, setacea_MP589, arachnocalyx_EO12453, muscosa_MP775, subcapitata_MP1042, passerinae_MP1302, exleeana_EO12499, condensata_MP890, anguliger_MP597, praecox_MP795, esterhuyseniae_EO11831, turgida_S1962, caterviflora_EO12785, bodkinii_TdV204, fillipendula_fil_MP914, gracilis_BG622, annectens_ANA, strigosa_MP673, trichophylla_EO10906, eremioides_MP533, canaliculata_BG590, adnata_MP501, bergiana_MP768, imbricata_MP688, haemastoma_MP871, benthamiana_MP560, glutinosa_MP687, floccifera_MP987, montis_hominis_EO11827, denticulata_MP799, phillipsii_MP794, discolor_heb_MP1214, fastigiata_MP830, corifolia_ANA, chiroptera_MP814, petrophila_EO7592, pannosa_EO12490, caespitosa_MP642, caledonica_JW103, cyrilliflora_CM10, longimontana_MP587, oblongiflora_ANA, simulans_ANA, curvifolia_MP700, brevifolia_EO12459, caprina_EO12772, colorans_EO12717, chamissonis_RT2188, diaphana_BG611, ocellata_MP574, sparrmanii_ANA, clavisepala_ANA, pubigera_MP572, dianthifolia_MP583, leucanthera_EO12452, halicacaba_ANA, nutans_BG599, placentiflora_EO12477, lepidota_MP541, collina_EO12613, corydalis_MP1005, parviflora_EO12492, ioniana_EO12781, regerminans_MP576, pycnantha_MP1011, spectabilis_MP929, tegulifolia_MP557, desmantha_MP562, zwartbergensis_MP608, dolfiana_MP1297, capensis_MP1047, ventricosa_MP713, krugeri_EO12807, fausta_MP663, atromontana_EO12544, karooica_MP1285, erinus_MP907, sphaerocephala_MP848, lucida_MP690, trichophora_EO12701, obtusata_EO12458, madida_MP573, rosacea_gla_BG628, rhodopis_BAB13, axillaris_MP1052, hanekomii_EO11172, steinbergiana_EO12763, transparens_MP893, georgica_ANA, magistrati_EO11750, urna_viridis_MP946, ampullacea_MP1277, phacelanthera_EO12489, cumuliflora_EO12699, oxycoccifolia_MP1275, grata_MP879, demissa_EO12540, copiosa_BG610, lambertii_ANA, triflora_MP564, malmesburiensis_EO12575, oresigena_MP759, multumbellifera_MP822, abietina_abi_MP1013, tetrathecoides_MP1252, eustacei_MP1259, mira_MP1257, ustulescens_RT1553, cristata_MP820, savileae_MP975, pubescens_EO12503, eburnea_MP1037, gibbosa_ANA, heleogena_MP1064, adunca_EO12746, phaeocarpa_SM2003, agglutinans_EO7679, caffrorum_MP644, holtii_TO, alopecurus_MP630, natalitia_EO12514, oatesii_ANA, cooperi_EO12588, woodii_DB1444, aspalathifolia_DB1408, reenensis_MP661 | | | | | | | | |
| --- | --- | --- | --- | --- | --- | --- | --- | --- |
| **Model** | **Dispersal multiplier** | **LnL** | **d [1/Ma]** | **e [1/Ma]** | **j** | **AIC** | **deltaAIC overall** | **deltaAIC per comparison** |
| no constraint |  | -58,35 | 0,0006 | 1,00E-12 | 0,0018 | 122,8 | 14,1 | 8,2 |
| Max area=2 |  | -58,28 | 6,00E-04 | 1,00E-12 | 0,0018 | 122,6 | 13,9 | 8 |
| Adjacency matrix |  | -54,24 | 2,40E-03 | 1,00E-12 | 0,0016 | 114,6 | 5,9 | 0 |
| Max area=2 + adjacency matrix |  | -54,24 | 2,40E-03 | 1,00E-12 | 0,0016 | 114,6 | 5,9 | 0 |
|  |  |  |  |  |  |  |  |  |
| **The following models are based on the best model above** | | | | | | | | |
| Stepping Stone (w=1) | 0 | -80,94 | 7,10E-03 | 1,00E-12 | 0,14 | 168 | 59,3 | 59,3 |
|  | 1 | -59,6 | 6,20E-03 | 1,00E-12 | 0,037 | 125,3 | 16,6 | 16,6 |
|  | 5 | -54,63 | 5,80E-03 | 1,00E-12 | 0,020 | 115,3 | 6,6 | 6,6 |
|  | 7,5 | -53,89 | 5,60E-03 | 1,00E-12 | 0,016 | 113,8 | 5,1 | 5,1 |
|  | 10 | -53,5 | 5,40E-03 | 1,00E-12 | 0,013 | 113,1 | 4,4 | 4,4 |
|  | 25 | -53,02 | 4,50E-03 | 1,00E-12 | 0,0061 | 112,1 | 3,4 | 3,4 |
|  | 50 | -53,36 | 3,50E-03 | 1,00E-12 | 0,0032 | 112,8 | 4,1 | 4,1 |
|  |  |  |  |  |  |  |  |  |
| Cape to Cairo (w=1) | 0 | -73,25 | 0,0053 | 0,0008 | 0,0084 | 152,6 | 43,9 | 43,9 |
|  | 1 | -60,09 | 2,60E-03 | 1,00E-12 | 0,0069 | 126,3 | 17,6 | 17,6 |
|  | 5 | -56,31 | 2,90E-03 | 1,00E-12 | 0,0061 | 118,7 | 10 | 10 |
|  | 7,5 | -55,47 | 2,90E-03 | 1,00E-12 | 0,0058 | 117 | 8,3 | 8,3 |
|  | 10 | -54,95 | 2,90E-03 | 1,00E-12 | 0,0054 | 116 | 7,3 | 7,3 |
|  | 25 | -53,81 | 2,80E-03 | 1,00E-12 | 0,0039 | 113,7 | 5 | 5 |
|  | 50 | -53,68 | 2,70E-03 | 1,00E-12 | 0,0027 | 113,4 | 4,7 | 4,7 |
|  |  |  |  |  |  |  |  |  |
| **Drakensberg Melting-pot (w=1)** | 0 | -60,23 | 0,0031 | 0,0008 | 0,0073 | 126,5 | 17,8 | 17,8 |
|  | 1 | -52,53 | 2,30E-03 | 1,00E-12 | 0,0066 | 111,1 | 2,4 | 2,4 |
|  | **5** | **-51,3** | **2,30E-03** | **1,00E-12** | **0,0053** | **108,7** | **0** | **0** |
|  | **7,5** | **-51,31** | **2,30E-03** | **1,00E-12** | **0,0056** | **108,7** | **0** | **0** |
|  | **10** | **-51,3** | **2,30E-03** | **1,00E-12** | **0,0053** | **108,7** | **0** | **0** |
|  | **25** | **-51,76** | **2,30E-03** | **1,00E-12** | **0,0039** | **109,6** | **0,9** | **0,9** |
| (E, aborea European) | 25 |  |  |  |  |  |  |  |
| w =0,8 | 25 |  |  |  |  |  |  |  |
| w =0,5 | 25 |  |  |  |  |  |  |  |
| w =0,1 | 25 |  |  |  |  |  |  |  |
|  | 50 | -52,73 | 0,0024 | 1,00E-12 | 0,0027 | 111,5 | 2,8 | 2,8 |
|  |  |  |  |  |  |  |  |  |
| **Geographic distance** | **As disp, probability (0 to 1)** | **-55,8** | **3,40E-03** | **1,00E-12** | **0,0026** | **117,7** | 9 | 3,9 |
|  | As distance (1 to x) | -64,81 | 3,00E-04 | 1,00E-12 | 1,60E-05 | 135,7 | 27 | 21,9 |
|  | As distance ^ -0,25 | -54,05 | 2,90E-03 | 1,00E-12 | 0,0033 | 114,2 | 5,5 | 0,4 |
|  | As distance ^ -1 | -60,23 | 0,0032 | 1,00E-12 | 0,0074 | 126,5 | 17,8 | 12,7 |
|  | As distance ^ -2 | -71,5 | 2,50E-03 | 1,00E-12 | 0,008 | 149,1 | 40,4 | 35,3 |
| Niche similarity | Schoener‘s D | -54,61 | 4,10E-03 | 1,00E-12 | 0,0032 | 115,3 | 6,6 | 1,5 |
| Niche plus distance as multipl, | As disp, probability/distance matrix | -53,86 | 4,20E-03 | 1,00E-12 | 0,0048 | 113,8 | 5,1 | 0 |

| Pruned tree 5 - deleted tips:  terminalis_a_ANA, umbellata_DS, carnea_ATsn5, spiculifolia_AS57234, scoparia_AH, mackayana_b_ANA, australis_b_ANA, erigena_a_ANA, ciliaris_c_ANA, lanceolifera_RC463, simii_RC466, trimera_MsnA, nematophylla_EO12747, nabea_ANA, orientalis_EO12608, monsoniana_MP986, jasminiflora_EO12612, loganii_MP1258, hansfordii_MP1239, dolfiana_MP1297, humifusa_MP846, bergiana_MP768, odorata_MP561, empetrina_EO12786, haemastoma_MP871, rhodopis_BAB13, remota_EO10386, pyxidiflora_ANA, viscaria_vis_MdV4, platycalyx_MP1243, strigilifolia_MP619, stagnalis_sta_MP668, conspicua_CS1, rubiginosa_RT1554, radicans_sch_MP1018, cruenta_MP745, pinea_MP789, lambertii_ANA, urceolata_MP955, fimbriata_MP606, ericoides_MP742, fillipendula_fil_MP914, similis_MP804, garciae_MP1253, clavisepala_ANA, phaeocarpa_SM2003, inflata_MP784, retorta_ANA, holosericea_EO12842, ardens_MP1076, chionodes_EO11699, placentiflora_EO12477, paucifolia_cil_EO12528, dregei_EO12711, incarnata_EO12771, bruniades_EO12465, daphniflora_MP567, intermedia_MM5082, albertyniae_MP927, podophylla_MP582, eriophoros_EO12478, lepidota_MP541, saxicola_EO12515, lavandulifolia_EO12506, leucodesmia_MP724, inordinata_EO11823, tetrathecoides_MP1252, nemerosa_MP1208, elimensis_EO12843, thimifolia_CM7, adnata_MP501, corydalis_MP1005, paniculata_MP1274, bodkinii_TdV204, barbigeroides_MP735, malmesburiensis_EO12575, tradouwensis_MP903, fascicularis_fac_MP809, magnisylvae_EO10708, glabella_la_EO11224, glandulipila_MP521, jonasiana_MP985, prolata_EO12748, astroites_EO12758, annectens_ANA, melanthera_MP610, lasciva_MP906, caprina_EO12772, vallis_aranearum_ANA, toringbergensis_ANA, uysii_ANA, passerinae_MP1302, atrovinosa_MP864, magistrati_EO11750, artemisioides_MP551, arcuata_MP523, sp_nov_MP1291, chionophila_MP790, massonii_MP811, vestita_EO12702, distorta_EO12500, thamnoides_MP1211, montis_hominis_EO11827, labialis_MP696, juniperina_SV952, planifolia_MP1012, globiceps_con_EO12519, fausta_MP663, hanekomii_EO11172, areolata_EO12502, gerhardii_EO12700, regerminans_MP576, margaritacea_ANA, walkeri_MP1237, perspicua_per_MP821, brachysepala_EO12727, haematocodon_MP1033, fontana_MP1069, laeta_MP1045, banksii_com_ANA, gracilipes_MM5014, zwartbergensis_MP608, caledonica_JW103, lithophila_MP1301, plukenetii_plu_EO, cumuliflora_EO12699, heleophila_ANA, hottentotica_EO, ventricosa_MP713, lehmannii_MP625, eburnea_MP1037, capensis_MP1047, canescens_ANA, umbratica_EO12760, phacelanthera_EO12489, glauca_gla_MP850, straussiana_MP638, hillburtii_EO12593, dissimulans_EO12596, atherstonei_EO12261, revoluta_BT13679, oatesii_ANA, caffrorum_MP644, woodii_DB1444, rivularis_BB13936 | | | | | | | | |
| --- | --- | --- | --- | --- | --- | --- | --- | --- |
| **Model** | **Dispersal multiplier** | **LnL** | **d [1/Ma]** | **e [1/Ma]** | **j** | **AIC** | **deltaAIC overall** | **deltaAIC per comparison** |
| no constraint |  | -63,02 | 0,0008 | 3,00e-10 | 0,0015 | 132,1 | 14,8 | 10,7 |
| Max area=2 |  | -62,95 | 8,00E-04 | 1,00E-12 | 0,0015 | 132 | 14,7 | 10,6 |
| Adjacency matrix |  | -57,66 | 3,20E-03 | 1,00E-12 | 0,0012 | 121,4 | 4,1 | 0 |
| Max area=2 + adjacency matrix |  | -57,66 | 3,20E-03 | 1,00E-12 | 0,0012 | 121,4 | 4,1 | 0 |
|  |  |  |  |  |  |  |  |  |
| **The following models are based on the best model above** | | | | | | | | |
| Stepping Stone (w=1) | 0 | -86,93 | 8,00E-03 | 1,00E-12 | 0,14 | 179,9 | 62,6 | 62,6 |
|  | 1 | -63,4 | 7,10E-03 | 1,00E-12 | 0,041 | 132,9 | 15,6 | 15,6 |
|  | 5 | -58,68 | 7,10E-03 | 1,00E-12 | 0,021 | 123,4 | 6,1 | 6,1 |
|  | 7,5 | -57,96 | 7,10E-03 | 1,00E-12 | 0,015 | 122 | 4,7 | 4,7 |
|  | 10 | -57,55 | 7,00E-03 | 1,00E-12 | 0,012 | 121,2 | 3,9 | 3,9 |
|  | 25 | -56,84 | 6,10E-03 | 1,00E-12 | 0,005 | 119,7 | 2,4 | 2,4 |
|  | 50 | -56,95 | 4,70E-03 | 1,00E-12 | 0,0025 | 120 | 2,7 | 2,7 |
|  |  |  |  |  |  |  |  |  |
| Cape to Cairo (w=1) | 0 | -65,25 | 0,0043 | 1,00E-12 | 0,0076 | 136,6 | 19,3 | 19,3 |
|  | 1 | -62,5 | 4,30E-03 | 1,00E-12 | 0,007 | 131,1 | 13,8 | 13,8 |
|  | 5 | -60,32 | 3,90E-03 | 1,00E-12 | 0,0055 | 126,7 | 9,4 | 9,4 |
|  | 7,5 | -59,59 | 3,90E-03 | 1,00E-12 | 0,005 | 125,2 | 7,9 | 7,9 |
|  | 10 | -59,07 | 3,80E-03 | 1,00E-12 | 0,0046 | 124,2 | 6,9 | 6,9 |
|  | 25 | -57,8 | 3,70E-03 | 1,00E-12 | 0,0032 | 121,7 | 4, | 4,4 |
|  | 50 | -57,41 | 3,50E-03 | 1,00E-12 | 0,0021 | 120,9 | 3,6 | 3,6 |
|  |  |  |  |  |  |  |  |  |
| **Drakensberg Melting-pot (w=1)** | 0 | -59,32 | 0,0033 | 1,00E-12 | 0,0075 | 124,7 | 7,4 | 7,4 |
|  | 1 | -56,93 | 3,00E-03 | 1,00E-12 | 0,006 | 119,9 | 2,6 | 2,6 |
|  | **5** | **-55,62** | **3,00E-03** | **1,00E-12** | **0,0045** | **117,3** | **0** | **0** |
|  | **7,5** | **-55,67** | **3,00E-03** | **1,00E-12** | **0,0048** | **117,4** | **0,1** | **0,1** |
|  | **10** | **-55,62** | **3,00E-03** | **1,00E-12** | **0,0045** | **117,3** | **0** | **0** |
|  | **25** | **-55,87** | **3,10E-03** | **1,00E-12** | **0,0032** | **117,8** | **0,5** | **0,5** |
| (E, aborea European) | 25 |  |  |  |  |  |  |  |
| w =0,8 | 25 |  |  |  |  |  |  |  |
| w =0,5 | 25 |  |  |  |  |  |  |  |
| w =0,1 | 25 |  |  |  |  |  |  |  |
|  | **50** | **-56,56** | **0,0031** | **1,00E-12** | **0,0021** | **119,2** | **1,9** | **1,9** |
|  |  |  |  |  |  |  |  |  |
| Geographic distance | As disp, probability (0 to 1) | -59,37 | 4,60E-03 | 1,00E-12 | 0,002 | 124,8 | 7,5 | 3,2 |
|  | As distance (1 to x) | -66,82 | 3,00E-04 | 1,00E-12 | 1,30E-05 | 139,7 | 22,4 | 18,1 |
|  | As distance ^ -0,25 | -57,9 | 3,80E-03 | 1,00E-12 | 0,0026 | 121,9 | 4,6 | 0,3 |
|  | As distance ^ -1 | -64,46 | 0,004 | 1,00E-12 | 0,0069 | 135 | 17,7 | 13,4 |
|  | As distance ^ -2 | -75,39 | 3,80E-03 | 1,00E-12 | 0,0062 | 156,9 | 39,6 | 35,3 |
| Niche similarity | Schoener‘s D | -58,2 | 5,40E-03 | 1,00E-12 | 0,0025 | 122,5 | 5,2 | 0,9 |
| Niche plus distance as multipl, | As disp, probability/distance matrix | -57,75 | 5,40E-03 | 1,00E-12 | 0,0039 | 121,6 | 4,3 | 0 |

| Pruned tree 6 - deleted tips:  umbellata_DS, sicula_sic_AM, erigena_a_ANA, cinerea_a_ANA, ciliaris_c_ANA, maderensis_AH, terminalis_a_ANA, scoparia_AH, australis_b_ANA, kingaensis_rug_BB2981, silvatica_A2, benguelensis_A1, rigidula_MP534, juniperina_SV952, cameronii_ANA, caledonica_JW103, amidae_EO12272, maderi_MP757, corydalis_MP1005, parviporandra_MP877, prolata_EO12748, georgica_ANA, fascicularis_fac_MP809, cyathiformis_a_ANA, placentiflora_EO12477, floccifera_MP987, melanthera_MP610, orientalis_EO12608, capitata_ANA, amoena_MP1032, deflexa_MP1247, oresigena_MP759, penduliflora_MP923, macowanii_mac_MP810, rosacea_gla_BG628, stylaris_ANA, accommodata_EO11382, aneimena_EO12757, shannonii_TdV262, thimifolia_CM7, conspicua_CS1, sonderiana_MP756, turgida_S1962, angulosa_S2105, loganii_MP1258, exleeana_EO12499, depressa_MP942, passerinae_MP1302, canaliculata_BG590, petrophila_EO7592, paniculata_MP1274, urceolata_MP955, hibbertii_MP982, cubica_MP623, paucifolia_cil_EO12528, pycnantha_MP1011, colorans_EO12717, nemerosa_MP1208, trichroma_EO12517, dispar_EO12749, stagnalis_sta_MP668, wendlandiana_EO12731, glutinosa_MP687, pageana_ANA, sparrmanii_ANA, labialis_MP696, leucanthera_EO12452, rusticula_EO12471, lateriflora_EO12482, selaginifolia_EO12488, trichophylla_EO10906, occulta_EO, hermani_EO12498, pubigera_MP572, nematophylla_EO12747, saxicola_EO12515, nudiflora_MP802, gracilis_BG622, corifolia_ANA, margaritacea_ANA, verecunda_CS5, regia_reg_MP922, leucodesmia_MP724, podophylla_MP582, bergiana_MP768, viscaria_vis_MdV4, melastoma_mel_MP773, montis_hominis_EO11827, oxycoccifolia_MP1275, astroites_EO12758, palliiflora_EO12533, pubescens_EO12503, marifolia_CM4, calycina_EO12532, cumuliflora_EO12699, bodkinii_TdV204, uberiflora_BG586, strigosa_MP673, setacea_MP589, bicolor_MP1098, stokoeanthus_EO4790, distorta_EO12500, monsoniana_MP986, pillansii_pil_MP813, perspicua_per_MP821, sparsa_BG602, fausta_MP663, odorata_MP561, banksii_com_ANA, artemisioides_MP551, chamissonis_RT2188, trichophora_EO12701, multumbellifera_MP822, cetrata_EO12064, alfredii_FR, rhodopis_BAB13, arachnocalyx_EO12453, collina_EO12613, inaequalis_MP539, bracteolaris_MP577, chionodes_EO11699, capensis_MP1047, cristata_MP820, karwyderi_EO12718, erinus_MP907, chartacea_EO11408, canescens_ANA, serrata_MP818, grata_MP879, copiosa_BG610, dodii_EO11417, laeta_MP1045, parilis_MP751, stokoei_MP825, multiflexuosa_EO12445, massonii_MP811, aspalathifolia_DB1408, cooperi_EO12588, thodei_MP656, atherstonei_EO12261, evansii_MP641, straussiana_MP638, oatesii_ANA, drakensbergensis_DB1443, swaziensis_L1187 | | | | | | | | |
| --- | --- | --- | --- | --- | --- | --- | --- | --- |
| **Model** | **Dispersal multiplier** | **LnL** | **d [1/Ma]** | **e [1/Ma]** | **j** | **AIC** | **deltaAIC overall** | **deltaAIC per comparison** |
| no constraint |  | -61,1 | 0,0007 | 1,0e-12 | 0,0015 | 128,3 | 14,7 | 10,2 |
| Max area=2 |  | -60,96 | 8,00E-04 | 1,00E-12 | 0,0015 | 128 | 14,4 | 9,9 |
| Adjacency matrix |  | -56,02 | 3,10E-03 | 1,00E-12 | 0,0012 | 118,1 | 4,5 | 0 |
| Max area=2 + adjacency matrix |  | -56,01 | 3,10E-03 | 1,00E-12 | 0,0012 | 118,1 | 4,5 | 0 |
|  |  |  |  |  |  |  |  |  |
| **The following models are based on the best model above** | | | | | | | | |
| Stepping Stone (w=1) | 0 | -84,03 | 7,80E-03 | 1,00E-12 | 0,16 | 174,1 | 60,5 | 60,5 |
|  | 1 | -61,5 | 7,20E-03 | 1,00E-12 | 0,041 | 129,1 | 15,5 | 15,5 |
|  | 5 | -56,85 | 7,10E-03 | 1,00E-12 | 0,021 | 119,8 | 6,2 | 6,2 |
|  | 7,5 | -56,16 | 7,00E-03 | 1,00E-12 | 0,015 | 118,4 | 4,8 | 4,8 |
|  | 10 | -55,78 | 6,90E-03 | 1,00E-12 | 0,012 | 117,6 | 4 | 4 |
|  | 25 | -55,15 | 6,00E-03 | 1,00E-12 | 0,0051 | 116,4 | 2,8 | 2,8 |
|  | 50 | -55,3 | 4,60E-03 | 1,00E-12 | 0,0025 | 116,7 | 3,1 | 3,1 |
|  |  |  |  |  |  |  |  |  |
| Cape to Cairo (w=1) | 0 | -72,37 | 4,10E-03 | 0,0008 | 0,010 | 150,8 | 37,2 | 37,2 |
|  | 1 | -60,99 | 3,10E-03 | 1,00E-12 | 0,0063 | 128 | 14,4 | 14,4 |
|  | 5 | -58 | 3,30E-03 | 1,00E-12 | 0,0055 | 122,1 | 8,5 | 8,5 |
|  | 7,5 | -57,34 | 3,30E-03 | 1,00E-12 | 0,0051 | 120,8 | 7,2 | 7,2 |
|  | 10 | -56,91 | 3,40E-03 | 1,00E-12 | 0,0047 | 119,9 | 6,3 | 6,3 |
|  | 25 | -55,91 | 3,50E-03 | 1,00E-12 | 0,0033 | 117,9 | 4,3 | 4,3 |
|  | 50 | -55,67 | 3,40E-03 | 1,00E-12 | 0,0022 | 117,4 | 3,8 | 3,8 |
|  |  |  |  |  |  |  |  |  |
| **Drakensberg Melting-pot (w=1)** | 0 | -62,86 | 3,70E-03 | 0,0008 | 0,007 | 131,8 | 18,2 | 18,2 |
|  | 1 | -55,04 | 2,80E-03 | 1,00E-12 | 0,0065 | 116,1 | 2,5 | 2,5 |
|  | **5** | **-53,76** | **2,90E-03** | **1,00E-12** | **0,0049** | **113,6** | **0** | **0** |
|  | **7,5** | **-53,79** | **2,80E-03** | **1,00E-12** | **0,0053** | **113,7** | **0,1** | **0,1** |
|  | **10** | **-53,76** | **2,90E-03** | **1,00E-12** | **0,0049** | **113,6** | **0** | **0** |
|  | **25** | **-54,1** | **3,00E-03** | **1,00E-12** | **0,0034** | **114,3** | **0,7** | **0,7** |
| (E, aborea European) | 25 |  |  |  |  |  |  |  |
| w =0,8 | 25 |  |  |  |  |  |  |  |
| w =0,5 | 25 |  |  |  |  |  |  |  |
| w =0,1 | 25 |  |  |  |  |  |  |  |
|  | 50 | -54,85 | 3,10E-03 | 1,00E-12 | 0,0022 | 115,8 | 2,2 | 2,2 |
|  |  |  |  |  |  |  |  |  |
| Geographic distance | As disp, probability (0 to 1) | -56,68 | 3,70E-03 | 1,00E-12 | 0,0022 | 119,4 | 5,8 | 1,5 |
|  | As distance (1 to x) | -65,1 | 3,00E-04 | 1,00E-12 | 1,30E-05 | 136,3 | 22,7 | 18,4 |
|  | As distance ^ -0,25 | -56,18 | 3,60E-03 | 1,00E-12 | 0,0026 | 118,4 | 4,8 | 0,5 |
|  | As distance ^ -1 | -61,84 | 3,60E-03 | 1,00E-12 | 0,006 | 129,8 | 16,2 | 11,9 |
|  | As distance ^ -2 | -71,55 | 3,50E-03 | 1,00E-12 | 0,0056 | 149,2 | 35,6 | 31,3 |
| Niche similarity | Schoener‘s D | -56,49 | 5,10E-03 | 1,00E-12 | 0,0025 | 119,1 | 5,5 | 1,2 |
| Niche plus distance as multipl, | As disp, probability/distance matrix | -55,93 | 5,00E-03 | 1,00E-12 | 0,004 | 117,9 | 4,3 | 0 |

| Pruned tree7 - deleted tips:  maderensis_AH, manipuliflora_a_ANA, ciliaris_c_ANA, terminalis_a_ANA, multiflora_a_ANA, umbellata_DS, erigena_a_ANA, sicula_sic_AM, mackayana_b_ANA, benguelensis_A1, kingaensis_rug_BB2981, microdonta_A5, blandfordii_MM4208, podophylla_MP582, cernua_EO12474, desmantha_MP562, stokoei_MP825, zwartbergensis_MP608, arachnocalyx_EO12453, fillipendula_fil_MP914, oreotragus_ANA, exleeana_EO12499, coarctata_ANA, plumigera_EO11341, umbratica_EO12760, patens_EO12457, eriophoros_EO12478, bergiana_MP768, arcuata_MP523, margaritacea_ANA, atromontana_EO12544, excavata_GK1532, parviflora_EO12492, inflata_MP784, lucida_MP690, pogonanthera_EO12835, perlata_MP960, maximilianii_EO12484, regerminans_MP576, bruniifolia_EO12460, corifolia_ANA, karooica_MP1285, maderi_MP757, hermani_EO12498, adunca_EO12746, pillansii_pil_MP813, incarnata_EO12771, petricola_MP996, penduliflora_MP923, anguliger_MP597, humidicola_EO11353, obtusata_EO12458, nudiflora_MP802, uberiflora_BG586, radicans_sch_MP1018, curvirostris_MP817, fontana_MP1069, areolata_EO12502, ocellata_MP574, melastoma_mel_MP773, pannosa_EO12490, madida_MP573, odorata_MP561, tradouwensis_MP903, hibbertii_MP982, curviflora_MP765, longimontana_MP587, strigosa_MP673, barbigeroides_MP735, velatiflora_EO12547, gibbosa_ANA, magnisylvae_EO10708, grata_MP879, platycalyx_MP1243, cetrata_EO12064, sparrmanii_ANA, petiolaris_EO12783, ericoides_MP742, spumosa_MP978, tomentosa_MP961, cruenta_MP745, chionodes_EO11699, blenna_ANA, distorta_EO12500, haematocodon_MP1033, tristis_MP932, tetrathecoides_MP1252, esterhuyseniae_EO11831, malmesburiensis_EO12575, alfredii_FR, cubica_MP623, chrysocodon_ANA, planifolia_MP1012, hansfordii_MP1239, interrupta_MP911, glutinosa_MP687, marifolia_CM4, rubiginosa_RT1554, glabella_la_EO11224, scytophylla_MP1021, bolusiae_ANA, physophylla_EO11418, paucifolia_cil_EO12528, viscaria_vis_MdV4, capillaris_MP1066, amidae_EO12272, dregei_EO12711, clavisepala_ANA, sociorum_cf_MP1055, elimensis_EO12843, ovina_EO12487, versicolor_ver_MP1232, astroites_EO12758, angulosa_S2105, viridiflora_MP1246, muscosa_MP775, squarrosa_EO11742, cyathiformis_a_ANA, glomiflora_EO12548, chartacea_EO11408, ventricosa_MP713, serrata_MP818, jonasiana_MP985, artemisioides_MP551, halicacaba_ANA, spectabilis_MP929, perspicua_per_MP821, paniculata_MP1274, globiceps_con_EO12519, caprina_EO12772, empetrina_EO12786, vestita_EO12702, phillipsii_MP794, macrotrema_MM4625, lambertii_ANA, verticillata_ANA, swaziensis_L1187, dominans_MP648, rivularis_BB13936, evansii_MP641, cooperi_EO12588, natalitia_EO12514, tysonii_EO12583, algida_MP645, thodei_MP656 | | | | | | | | |
| --- | --- | --- | --- | --- | --- | --- | --- | --- |
| **Model** | **Dispersal multiplier** | **LnL** | **d [1/Ma]** | **e [1/Ma]** | **j** | **AIC** | **deltaAIC overall** | **deltaAIC per comparison** |
| no constraint |  | -62.63 | 0.0008 | 1,0e-12 | 0,0015 | 131.3 | 15.2 | 10.9 |
| Max area=2 |  | -62,54 | 0,0008 | 1,0e-12 | 0,0015 | 131,1 | 14,9 | 10,7 |
| Adjacency matrix |  | -57,2 | 0,0032 | 1,0e-12 | 0,0012 | 120,5 | 4,3 | 0,1 |
| Max area=2 + adjacency matrix |  | -57,19 | 0,0032 | 1,0e-12 | 0,0012 | 120,4 | 4,2 | 0 |
|  |  |  |  |  |  |  |  |  |
| **The following models are based on the best model above** | | | | | | | | |
| Stepping Stone (w=1) | 0 | -84,96 | 0,0074 | 1,0e-12 | 0,16 | 176 | 59,8 | 59,8 |
|  | 1 | -62,65 | 0,0069 | 1,0e-12 | 0,043 | 131,4 | 15,2 | 15,2 |
|  | 5 | -58,07 | 0,0069 | 1,0e-12 | 0,021 | 122,2 | 6 | 6 |
|  | 7,5 | -57,39 | 0,0069 | 1,0e-12 | 0,016 | 120,9 | 4,7 | 4,7 |
|  | 10 | -57,02 | 0,0068 | 1,0e-12 | 0,012 | 120,1 | 3,9 | 3,9 |
|  | 25 | -56,38 | 0,0060 | 1,0e-12 | 0,0050 | 118,8 | 2,6 | 2,6 |
|  | 50 | -56,5 | 0,0047 | 1,0e-12 | 0,0024 | 119,1 | 2,9 | 2,9 |
|  |  |  |  |  |  |  |  |  |
| Cape to Cairo (w=1) | 0 | -74,18 | 0,0049 | 0,0008 | 0,0096 | 154,4 | 38,2 | 38,2 |
|  | 1 | -63,92 | 0,0035 | 1,0e-12 | 0,0062 | 133,9 | 17,7 | 17,7 |
|  | 5 | -60,16 | 0,0037 | 1,0e-12 | 0,0053 | 126,4 | 10,2 | 10,2 |
|  | 7,5 | -59,29 | 0,0038 | 1,0e-12 | 0,0049 | 124,6 | 8,4 | 8,4 |
|  | 10 | -58,73 | 0,0038 | 1,0e-12 | 0,0045 | 123,5 | 7,3 | 7,3 |
|  | 25 | -57,39 | 0,0037 | 1,0e-12 | 0,0032 | 120,9 | 4,7 | 4,7 |
|  | 50 | -56,98 | 0,0036 | 1,0e-12 | 0,0020 | 120 | 3,8 | 3,8 |
|  |  |  |  |  |  |  |  |  |
| **Drakensberg Melting-pot (w=1)** | 0 | -63,76 | 0,0036 | 0,0008 | 0,0072 | 133,6 | 17,4 | 17,4 |
|  | 1 | -56,35 | 0,0028 | 1,0e-12 | 0,0066 | 118,8 | 2,6 | 2,6 |
|  | **5** | **-55,07** | **0,0029** | **1,0e-12** | **0,0049** | **116,2** | **0** | **0** |
|  | **7,5** | **-55,11** | **0,0029** | **1,0e-12** | **0,0053** | **116,3** | **0,1** | **0,1** |
|  | **10** | **-55,07** | **0,0029** | **1,0e-12** | **0,0049** | **116,2** | **0** | **0** |
|  | **25** | **-55,4** | **0,0030** | **1,0e-12** | **0,0033** | **116,9** | **0,7** | **0,7** |
| (E, aborea European) | 25 |  |  |  |  |  |  |  |
| w =0,8 | 25 |  |  |  |  |  |  |  |
| w =0,5 | 25 |  |  |  |  |  |  |  |
| w =0,1 | 25 |  |  |  |  |  |  |  |
|  | 50 | -56,11 | 0,0031 | 1,0e-12 | 0,0021 | 118,3 | 2,1 | 2,1 |
|  |  |  |  |  |  |  |  |  |
| Geographic distance | As disp, probability (0 to 1) | -58,85 | 0,0045 | 1,0e-12 | 0,0019 | 123,8 | 7,6 | 3,1 |
|  | As distance (1 to x) | -66,16 | 0,0003 | 1,0e-12 | 1,2e-05 | 138,4 | 22,2 | 17,7 |
|  | As distance ^ -0,25 | -57,5 | 0,0038 | 1,0e-12 | 0,0025 | 121,1 | 4,9 | 0,4 |
|  | As distance ^ -1 | -64,28 | 0,0042 | 1,0e-12 | 0,0057 | 134,6 | 18,4 | 13,9 |
|  | As distance ^ -2 | -75,05 | 0,0036 | 1,0e-12 | 0,0054 | 156,2 | 40 | 35,5 |
| Niche similarity | Schoener‘s D | -57,76 | 0,0054 | 1,0e-12 | 0,0024 | 121,6 | 5,4 | 0,9 |
| Niche plus distance as multipl, | As disp, probability/distance matrix | -57,34 | 0,0054 | 1,0e-12 | 0,0037 | 120,7 | 4,5 | 0 |

| Pruned tree8 - deleted tips:  spiculifolia_AS57234, sicula_sic_AM, multiflora_a_ANA, ciliaris_c_ANA, umbellata_DS, carnea_ATsn5, mackayana_b_ANA, australis_b_ANA, scoparia_AH, benguelensis_A1, filago_BG93, hexandra_RC465, patersonii_a_ANA, tetrathecoides_MP1252, selaginifolia_EO12488, monsoniana_MP986, rubiginosa_RT1554, similis_MP804, caterviflora_EO12785, plukenetii_plu_EO, sitiens_MP827, longimontana_MP587, bolusiae_ANA, coacervata_MP761, karooica_MP1285, planifolia_MP1012, lasciva_MP906, umbelliflora_RT2182, colorans_EO12717, zwartbergensis_MP608, hirtiflora_MP958, zeyheriana_EO, daphniflora_MP567, bergiana_MP768, oxysepala_MP780, eriophoros_EO12478, scytophylla_MP1021, verecunda_CS5, retorta_ANA, setacea_MP589, ventricosa_MP713, lucida_MP690, modesta_ANA, hispidula_MP801, subcapitata_MP1042, curviflora_MP765, esterhuyseniae_EO11831, pannosa_EO12490, hibbertii_MP982, salteri_S2065, nana_ANA, greyi_EO12501, shannonii_TdV262, goatcheriana_dra_EO12694, oreotragus_ANA, hermani_EO12498, lateriflora_EO12482, ferrea_EO12494, albens_ANA, stagnalis_sta_MP668, erinus_MP907, parilis_MP751, peltata_MP1231, fimbriata_MP606, canescens_ANA, leucanthera_EO12452, rusticula_EO12471, pinea_MP789, toringbergensis_ANA, chartacea_EO11408, madida_MP573, cubica_MP623, lateralis_MP721, palliiflora_EO12533, stokoeanthus_EO4790, chionophila_MP790, serrata_MP818, viscaria_vis_MdV4, lavandulifolia_EO12506, squarrosa_EO11742, baccans_BG645, globiceps_con_EO12519, macowanii_mac_MP810, albescens_MP898, flacca_MP840, leucotrachela_ANA, lepidota_MP541, pogonanthera_EO12835, rugata_EO12516, caespitosa_MP642, georgica_ANA, caprina_EO12772, wendlandiana_EO12731, multiflexuosa_EO12445, nervata_EO12541, rubens_EO12479, magistrati_EO11750, thamnoides_MP1211, melastoma_mel_MP773, curtophylla_EO12750, albertyniae_MP927, saxicola_EO12515, uysii_ANA, glabella_la_EO11224, propendens_EO12464, villosa_EO11394, gibbosa_ANA, ecklonii_EO12739, pillansii_pil_MP813, aneimena_EO12757, maximilianii_EO12484, steinbergiana_EO12763, nemerosa_MP1208, corifolia_ANA, gerhardii_EO12700, atromontana_EO12544, phacelanthera_EO12489, gracilipes_MM5014, sparrmanii_ANA, trichophora_EO12701, recurvifolia_EO12475a, simulans_ANA, leonis_RTsn, remota_EO10386, intermedia_MM5082, regerminans_MP576, tristis_MP932, cereris_MP863, ocellata_MP574, monadelphia_FO, chionodes_EO11699, nubigena_MP868, nevillei_MP1056, oliveri_MP1278, floccifera_MP987, multumbellifera_MP822, drakensbergensis_DB1443, leucopelta_EO12598, aspalathifolia_DB1408, thodei_MP656, tysonii_EO12583, psittacina_IJ1237, evansii_MP641, reenensis_MP661, natalitia_EO12514 | | | | | | | | |
| --- | --- | --- | --- | --- | --- | --- | --- | --- |
| **Model** | **Dispersal multiplier** | **LnL** | **d [1/Ma]** | **e [1/Ma]** | **j** | **AIC** | **deltaAIC overall** | **deltaAIC per comparison** |
| no constraint |  | -55,6 | 0,0008 | 1,0e-12 | 0,0011 | 117,3 | 12,5 | 10 |
| Max area=2 |  | -55,48 | 0,0008 | 1,0e-12 | 0,0011 | 117 | 12,2 | 9,7 |
| Adjacency matrix |  | -50,61 | 0,0028 | 1,0e-12 | 0,0010 | 107,3 | 2,5 | 0 |
| Max area=2 + adjacency matrix |  | -50,6 | 0,0028 | 1,0e-12 | 0,0010 | 107,3 | 2,5 | 0 |
|  |  |  |  |  |  |  |  |  |
| **The following models are based on the best model above** | | | | | | | | |
| **Stepping Stone (w=1)** | 0 | -66,34 | 0,0088 | 1,0e-12 | 0,028 | 138,7 | 33,9 | 33,9 |
|  | 1 | -54,6 | 0,0071 | 1,0e-12 | 0,030 | 115,3 | 10,5 | 10,5 |
|  | 5 | -50,87 | 0,0070 | 1,0e-12 | 0,015 | 107,8 | 3 | 3 |
|  | 7,5 | -50,3 | 0,0069 | 1,0e-12 | 0,011 | 106,7 | 1,9 | 1,9 |
|  | 10 | -49,99 | 0,0067 | 1,0e-12 | 0,0088 | 106 | 1,2 | 1,2 |
|  | **25** | **-49,52** | **0,0056** | **1,0e-12** | **0,0038** | **105,1** | **0,3** | **0,3** |
|  | **50** | **-49,79** | **0,0042** | **1,0e-12** | **0,0019** | **105,6** | **0,8** | **0,8** |
|  |  |  |  |  |  |  |  |  |
| Cape to Cairo (w=1) | 0 | -69,05 | 0,0045 | 0,0008 | 0,0081 | 144,2 | 39,4 | 39,4 |
|  | 1 | -57,62 | 0,0031 | 1,0e-12 | 0,0046 | 121,3 | 16,5 | 16,5 |
|  | 5 | -54,23 | 0,0034 | 1,0e-12 | 0,0040 | 114,5 | 9,7 | 9,7 |
|  | 7,5 | -53,4 | 0,0034 | 1,0e-12 | 0,0037 | 112,9 | 8,1 | 8,1 |
|  | 10 | -52,84 | 0,0034 | 1,0e-12 | 0,0035 | 111,8 | 7 | 7 |
|  | 25 | -51,37 | 0,0034 | 1,0e-12 | 0,0025 | 108,8 | 4 | 4 |
|  | 50 | -50,72 | 0,0032 | 1,0e-12 | 0,0016 | 107,5 | 2,7 | 2,7 |
|  |  |  |  |  |  |  |  |  |
| **Drakensberg Melting-pot (w=1)** | 0 | -58,64 | 0,0036 | 0,0008 | 0,0054 | 123,4 | 18,6 | 18,6 |
|  | 1 | -50,89 | 0,0028 | 1,0e-12 | 0,0049 | 107,9 | 3,1 | 3,1 |
|  | **5** | **-49,37** | **0,0028** | **1,0e-12** | **0,0036** | **104,8** | **0** | **0** |
|  | **7,5** | **-49,46** | **0,0028** | **1,0e-12** | **0,0039** | **105** | **0,2** | **0,2** |
|  | **10** | **-49,37** | **0,0028** | **1,0e-12** | **0,0036** | **104,8** | **0** | **0** |
|  | **25** | **-49,4** | **0,0029** | **1,0e-12** | **0,0025** | **104,9** | **0,1** | **0,1** |
| (E, aborea European) | 25 |  |  |  |  |  |  |  |
| w =0,8 | 25 |  |  |  |  |  |  |  |
| w =0,5 | 25 |  |  |  |  |  |  |  |
| w =0,1 | 25 |  |  |  |  |  |  |  |
|  | **50** | **-49,82** | **0,0029** | **1,0e-12** | **0,0017** | **105,7** | **0,9** | **0,9** |
|  |  |  |  |  |  |  |  |  |
| Geographic distance | As disp, probability (0 to 1) | -52,04 | 0,0037 | 1,0e-12 | 0,0016 | 110,1 | 5,3 | 1,6 |
|  | As distance (1 to x) | -56,74 | 0,0003 | 1,0e-12 | 1,2e-05 | 119,5 | 14,7 | 11 |
|  | As distance ^ -0,25 | -51,36 | 0,0035 | 1,0e-12 | 0,0020 | 108,8 | 4 | 0,3 |
|  | As distance ^ -1 | -58,24 | 0,0037 | 1,0e-12 | 0,0043 | 122,6 | 17,8 | 14,1 |
|  | As distance ^ -2 | -68,18 | 0,0033 | 1,0e-12 | 0,0039 | 142,4 | 37,6 | 33,9 |
| Niche similarity | Schoener‘s D | -51,41 | 0,0048 | 1,0e-12 | 0,0019 | 108,9 | 4,1 | 0,4 |
| Niche plus distance as multipl, | As disp, probability/distance matrix | -51,2 | 0,0049 | 1,0e-12 | 0,0029 | 108,5 | 3,7 | 0 |

| Pruned tree9 - deleted tips:  vagans_MP972, mackayana_b_ANA, spiculifolia_AS57234, cinerea_a_ANA, tetralix_c_ANA, terminalis_a_ANA, erigena_a_ANA, maderensis_AH, scoparia_AH, whyteana_A4, lanceolifera_RC463, johnstoniana_RC464, dregei_EO12711, villosa_EO11394, bokkeveldia_EO12769, coarctata_MP590, chiroptera_MP814, petiolaris_EO12783, nutans_BG599, odorata_MP561, heleophila_ANA, gracilis_BG622, ardens_MP1076, rusticula_EO12471, calcareophila_EO30159, astroites_EO12758, paucifolia_cil_EO12528, umbelliflora_RT2182, rubiginosa_RT1554, perplexa_EO12788, sitiens_MP827, blenna_ANA, cetrata_EO12064, karooica_MP1285, sphaerocephala_MP848, stylaris_ANA, hendricksei_EO12524, patens_EO12457, praecox_MP795, hottentotica_EO, areolata_EO12502, ustulescens_RT1553, wendlandiana_EO12731, nana_ANA, vernicosa_MP928, uberiflora_BG586, haematocodon_MP1033, russakiana_MP684, colorans_EO12717, lambertii_ANA, pubescens_EO12503, discolor_heb_MP1214, sparrmanii_ANA, cylindrica_MP1240, cruenta_MP745, argentea_EO12475, recurvifolia_EO12475a, leucanthera_EO12452, laeta_MP1045, orientalis_EO12608, trichroma_EO12517, baueri_bau_MP1233, tradouwensis_MP903, gibbosa_ANA, multumbellifera_MP822, caledonica_JW103, chionodes_EO11699, zwartbergensis_MP608, phacelanthera_EO12489, massonii_MP811, dianthifolia_MP583, subdivaricata_MP671, oblongiflora_ANA, equisetifolia_MP829, serrata_MP818, caterviflora_EO12785, perlata_MP960, spectabilis_MP929, cernua_EO12474, umbratica_EO12760, uysii_ANA, lateralis_MP721, petrophila_EO7592, eburnea_MP1037, aneimena_EO12757, stagnalis_sta_MP668, stokoeanthus_EO4790, passerinae_MP1302, tomentosa_MP961, unilateralis_MP1205, obtusata_EO12458, lignosa_EO11763, lepidota_MP541, esterhuyseniae_EO11831, phillipsii_MP794, intermedia_MM5082, magistrati_EO11750, alfredii_FR, dolfiana_MP1297, tumida_MP755, pycnantha_MP1011, rubens_EO12479, prolata_EO12748, oxysepala_MP780, inordinata_EO11823, curviflora_MP765, sessiliflora_MP604, brevifolia_EO12459, transparens_MP893, accommodata_EO11382, viridiflora_MP1246, walkeri_MP1237, eriophoros_EO12478, abietina_abi_MP1013, bergiana_MP768, krugeri_EO12807, melanthera_MP610, interrupta_MP911, erinus_MP907, verecunda_CS5, nematophylla_EO12747, sp_nov_MP1291, jasminiflora_EO12612, leptopus_ANA, oligantha_TdV86, cristata_MP820, amidae_EO12272, placentiflora_EO12477, desmantha_MP562, axillaris_MP1052, pulchella_MP736, maderi_MP757, macowanii_mac_MP810, maximilianii_EO12484, curvirostris_MP817, dodii_EO11417, drakensbergensis_DB1443, cooperi_EO12588, reenensis_MP661, psittacina_IJ1237, atherstonei_EO12261, natalitia_EO12514, holtii_TO, thodei_MP656, swaziensis_L1187 | | | | | | | | |
| --- | --- | --- | --- | --- | --- | --- | --- | --- |
| **Model** | **Dispersal multiplier** | **LnL** | **d [1/Ma]** | **e [1/Ma]** | **j** | **AIC** | **deltaAIC overall** | **deltaAIC per comparison** |
| no constraint |  | -58,8 | 0,0006 | 1,0e-12 | 0,0018 | 123,7 | 14,2 | 8,4 |
| Max area=2 |  | -58,74 | 0,0006 | 1,0e-12 | 0,0018 | 123,6 | 14,1 | 8,3 |
| Adjacency matrix |  | -54,63 | 0,0024 | 1,0e-12 | 0,0015 | 115,3 | 5,8 | 0 |
| Max area=2 + adjacency matrix |  | -54,63 | 0,0024 | 1,0e-12 | 0,0015 | 115,3 | 5,8 | 0 |
|  |  |  |  |  |  |  |  |  |
| **The following models are based on the best model above** | | | | | | | | |
| Stepping Stone (w=1) | 0 | -84,59 | 1,10E+01 | 1,0e-12 | 0,045 | 175,2 | 65,7 | 65,7 |
|  | 1 | -59,81 | 0,0064 | 1,0e-12 | 0,036 | 125,7 | 16,2 | 16,2 |
|  | 5 | -54,95 | 0,0059 | 1,0e-12 | 0,020 | 116 | 6,5 | 6,5 |
|  | 7,5 | -54,22 | 0,0057 | 1,0e-12 | 0,016 | 114,5 | 5 | 5 |
|  | 10 | -53,85 | 0,0055 | 1,0e-12 | 0,013 | 113,8 | 4,3 | 4,3 |
|  | 25 | -53,39 | 0,0045 | 1,0e-12 | 0,0060 | 112,9 | 3,4 | 3,4 |
|  | 50 | -53,74 | 0,0035 | 1,0e-12 | 0,0031 | 113,6 | 4,1 | 4,1 |
|  |  |  |  |  |  |  |  |  |
| Cape to Cairo (w=1) | 0 | -70,02 | 0,0047 | 0,0007 | 0,0087 | 146,1 | 36,6 | 36,6 |
|  | 1 | -60,88 | 0,0029 | 1,0e-12 | 0,0071 | 127,8 | 18,3 | 18,3 |
|  | 5 | -56,85 | 0,0029 | 1,0e-12 | 0,0062 | 119,8 | 10,3 | 10,3 |
|  | 7,5 | -55,98 | 0,0029 | 1,0e-12 | 0,0058 | 118 | 8,5 | 8,5 |
|  | 10 | -55,44 | 0,0029 | 1,0e-12 | 0,0054 | 116,9 | 7,4 | 7,4 |
|  | 25 | -54,27 | 0,0028 | 1,0e-12 | 0,0040 | 114,6 | 5,1 | 5,1 |
|  | 50 | -54,11 | 0,0027 | 1,0e-12 | 0,0027 | 114,3 | 4,8 | 4,8 |
|  |  |  |  |  |  |  |  |  |
| **Drakensberg Melting-pot (w=1)** | 0 | -60,52 | 0,0031 | 0,0008 | 0,0074 | 127,1 | 17,6 | 17,6 |
|  | 1 | -52,94 | 0,0023 | 1,0e-12 | 0,0067 | 112 | 2,5 | 2,5 |
|  | **5** | **-51,71** | **0,0023** | **1,0e-12** | **0,0053** | **109,5** | **0** | **0** |
|  | **7,5** | **-51,73** | **0,0023** | **1,0e-12** | **0,0057** | **109,5** | **0** | **0** |
|  | **10** | **-51,71** | **0,0023** | **1,0e-12** | **0,0053** | **109,5** | **0** | **0** |
|  | **25** | **-52,18** | **0,0023** | **1,0e-12** | **0,0039** | **110,4** | **0,9** | **0,9** |
| (E, aborea European) | 25 |  |  |  |  |  |  |  |
| w =0,8 | 25 |  |  |  |  |  |  |  |
| w =0,5 | 25 |  |  |  |  |  |  |  |
| w =0,1 | 25 |  |  |  |  |  |  |  |
|  | **50** | **-53,15** | **0,0023** | **1,0e-12** | **0,0027** | **112,4** | 2,9 | 2,9 |
|  |  |  |  |  |  |  |  |  |
| Geographic distance | As disp, probability (0 to 1) | -56,38 | 0,0035 | 1,0e-12 | 0,0026 | 118,8 | 9,3 | 4,1 |
|  | As distance (1 to x) | -64,34 | 0,0003 | 7,0e-11 | 1,3e-05 | 1,35E+02 | 25,2 | 20 |
|  | As distance ^ -0,25 | -54,5 | 0,0029 | 1,0e-12 | 0,0032 | 115,1 | 5,6 | 0,4 |
|  | As distance ^ -1 | -60,85 | 0,0034 | 1,0e-12 | 0,0072 | 127,8 | 18,3 | 13,1 |
|  | As distance ^ -2 | -72,93 | 0,0030 | 1,0e-12 | 0,0070 | 151,9 | 42,4 | 37,2 |
| Niche similarity | Schoener‘s D | -55,05 | 0,0041 | 1,0e-12 | 0,0031 | 116,2 | 6,7 | 1,5 |
| Niche plus distance as multipl, | As disp, probability/distance matrix | -54,33 | 0,0041 | 1,0e-12 | 0,0047 | 114,7 | 5,2 | 0 |

| Pruned tree10 - deleted tips:  spiculifolia_AS57234, tetralix_c_ANA, sicula_sic_AM, erigena_a_ANA, carnea_ATsn5, vagans_MP972, multiflora_a_ANA, umbellata_DS, cinerea_a_ANA, thomensis_EO12615, silvatica_A2, simii_RC466, verecunda_CS5, vallis_fluminis_EO12761, orientalis_EO12608, velatiflora_EO12547, maximilianii_EO12484, distorta_EO12500, bolusiae_ANA, bicolor_MP1098, nabea_ANA, planifolia_MP1012, dolfiana_MP1297, stagnalis_sta_MP668, adunca_EO12746, glutinosa_MP687, sonderiana_MP756, garciae_MP1253, jasminiflora_EO12612, nubigena_MP868, physodes_ANA, albescens_MP898, corifolia_ANA, glandulipila_MP521, amidae_EO12272, obtusata_EO12458, sessiliflora_MP604, blandfordii_MM4208, caledonica_JW103, macrotrema_MM4625, multiflexuosa_EO12445, argentea_EO12475, leptopus_ANA, brachialis_MP734, selaginifolia_EO12488, wendlandiana_EO12731, polycoma_FR, nudiflora_MP802, perlata_MP960, setacea_MP589, bruniifolia_EO12460, parilis_MP751, oligantha_TdV86, capensis_MP1047, pageana_ANA, greyi_EO12501, chartacea_EO11408, interrupta_MP911, pannosa_EO12490, angulosa_S2105, infundibuliformis_MP1238, capitata_ANA, equisetifolia_MP829, lanata_MP1220, heleophila_ANA, vernicosa_MP928, dregei_EO12711, unicolor_uni_MP1249, pulvinata_MP1304, tegulifolia_MP557, saxicola_EO12515, hibbertii_MP982, bodkinii_TdV204, vallis_aranearum_ANA, stylaris_ANA, gracilipes_MM5014, riparia_MP908, simulans_ANA, podophylla_MP582, shannonii_TdV262, subcapitata_MP1042, incarnata_EO12771, prolata_EO12748, trichophylla_EO10906, pinea_MP789, hansfordii_MP1239, odorata_MP561, canescens_ANA, remota_EO10386, cristata_MP820, adnata_MP501, eremioides_MP533, cernua_EO12474, esterhuyseniae_EO11831, agglutinans_EO7679, goatcheriana_dra_EO12694, junonia_min_MP866, tomentosa_MP961, glomiflora_EO12548, rubiginosa_RT1554, fimbriata_MP606, propendens_EO12464, bracteolaris_MP577, juniperina_SV952, florifera_EO12536, paniculata_MP1274, loganii_MP1258, ustulescens_RT1553, chionodes_EO11699, fascicularis_fac_MP809, perplexa_EO12788, physantha_MP1242, trichroma_EO12517, lignosa_EO11763, densifolia_BG591, sparsa_BG602, acuta_MP506, curviflora_MP765, maderi_MP757, sp_pachysa_EO12720, hispiduloides_EO11544, duthieae_ANA, mira_MP1257, calcareophila_EO30159, coacervata_MP761, intermedia_MM5082, laeta_MP1045, curvirostris_MP817, copiosa_BG610, viscaria_vis_MdV4, similis_MP804, arachnocalyx_EO12453, hanekomii_EO11172, oxycoccifolia_MP1275, chiroptera_MP814, serrata_MP818, dominans_MP648, hillburtii_EO12593, algida_MP645, subverticillaris_EO12625, swaziensis_L1187, thodei_MP656, aspalathifolia_DB1408, caffrorum_MP644, glaphyra_MP647 | | | | | | | | |
| --- | --- | --- | --- | --- | --- | --- | --- | --- |
| **Model** | **Dispersal multiplier** | **LnL** | **d [1/Ma]** | **e [1/Ma]** | **j** | **AIC** | **deltaAIC overall** | **deltaAIC per comparison** |
| no constraint |  | -59,56 | 0,0006 | 1,0e-12 | 0,0018 | 125,2 | 14,2 | 8,2 |
| Max area=2 |  | -59,5 | 0,0006 | 1,0e-12 | 0,0018 | 125,1 | 14,1 | 8,1 |
| Adjacency matrix |  | -55,48 | 0,0024 | 1,0e-12 | 0,0016 | 117 | 6 | 0 |
| Max area=2 + adjacency matrix |  | -55,48 | 0,0024 | 1,0e-12 | 0,0016 | 117 | 6 | 0 |
|  |  |  |  |  |  |  |  |  |
| **The following models are based on the best model above** | | | | | | | | |
| Stepping Stone (w=1) | 0 | -84,52 | 1,10E+01 | 1,0e-12 | 0,054 | 175,1 | 64,1 | 64,1 |
|  | 1 | -60,62 | 0,0065 | 1,0e-12 | 0,037 | 127,3 | 16,3 | 16,3 |
|  | 5 | -55,71 | 0,0061 | 1,0e-12 | 0,020 | 117,5 | 6,5 | 6,5 |
|  | 7,5 | -54,99 | 0,0059 | 1,0e-12 | 0,016 | 116 | 5 | 5 |
|  | 10 | -54,61 | 0,0056 | 1,0e-12 | 0,013 | 115,3 | 4,3 | 4,3 |
|  | 25 | -54,18 | 0,0046 | 1,0e-12 | 0,0062 | 114,4 | 3,4 | 3,4 |
|  | 50 | -54,56 | 0,0036 | 1,0e-12 | 0,0032 | 115,2 | 4,2 | 4,2 |
|  |  |  |  |  |  |  |  |  |
| Cape to Cairo (w=1) | 0 | -74,41 | 0,0054 | 0,0008 | 0,0084 | 154,9 | 43,9 | 43,9 |
|  | 1 | -61,12 | 0,0026 | 1,0e-12 | 0,0070 | 128,3 | 17,3 | 17,3 |
|  | 5 | -57,46 | 0,0029 | 1,0e-12 | 0,0062 | 121 | 10 | 10 |
|  | 7,5 | -56,64 | 0,0029 | 1,0e-12 | 0,0058 | 119,4 | 8,4 | 8,4 |
|  | 10 | -56,12 | 0,0029 | 1,0e-12 | 0,0055 | 118,3 | 7,3 | 7,3 |
|  | 25 | -55,01 | 0,0029 | 1,0e-12 | 0,0040 | 116,1 | 5,1 | 5,1 |
|  | 50 | -54,89 | 0,0027 | 1,0e-12 | 0,0027 | 115,9 | 4,9 | 4,9 |
|  |  |  |  |  |  |  |  |  |
| **Drakensberg Melting-pot (w=1)** | 0 | -61,23 | 0,0032 | 0,0008 | 0,0074 | 128,5 | 17,5 | 17,5 |
|  | 1 | -53,7 | 0,0023 | 1,0e-12 | 0,0067 | 113,5 | 2,5 | 2,5 |
|  | **5** | **-52,47** | **0,0024** | **1,0e-12** | **0,0054** | **111** | **0** | **0** |
|  | **7,5** | **-52,49** | **0,0024** | **1,0e-12** | **0,0057** | **111** | **0** | **0** |
|  | **10** | **-52,47** | **0,0024** | **1,0e-12** | **0,0054** | **111** | **0** | **0** |
|  | **25** | **-52,95** | **0,0024** | **1,0e-12** | **0,0040** | **112** | **1** | **1** |
| (E, aborea European) | 25 |  |  |  |  |  |  |  |
| w =0,8 | 25 |  |  |  |  |  |  |  |
| w =0,5 | 25 |  |  |  |  |  |  |  |
| w =0,1 | 25 |  |  |  |  |  |  |  |
|  | 50 | -53,94 | 0,0024 | 1,0e-12 | 0,0027 | 114 | 3 | 3 |
|  |  |  |  |  |  |  |  |  |
| Geographic distance | As disp, probability (0 to 1) | -56,98 | 0,0033 | 1,0e-12 | 0,0027 | 120 | 9 | 3,7 |
|  | As distance (1 to x) | -66,15 | 0,0003 | 1,0e-12 | 1,8e-05 | 138,4 | 27,4 | 22,1 |
|  | As distance ^ -0,25 | -55,29 | 0,0029 | 1,0e-12 | 0,0033 | 116,6 | 5,6 | 0,3 |
|  | As distance ^ -1 | -61,4 | 0,0031 | 1,0e-12 | 0,0075 | 128,9 | 17,9 | 12,6 |
|  | As distance ^ -2 | -72,43 | 0,0025 | 1,0e-12 | 0,0080 | 150,9 | 39,9 | 34,6 |
| Niche similarity | Schoener‘s D | -55,87 | 0,0041 | 1,0e-12 | 0,0033 | 117,8 | 6,8 | 1,5 |
| Niche plus distance as multipl, | As disp, probability/distance matrix | -55,1 | 0,0042 | 1,0e-12 | 0,0048 | 116,3 | 5,3 | 0 |
