# Supplementary material for: Leaps and bounds: geographical and ecological distance constrained the colonisation of the Afrotemperate by Erica
Source: BMC Evol Biol. 2019 Dec 5;19:222. doi: 10.1186/s12862-019-1545-6 (PMC6896773; doi:10.1186/s12862-019-1545-6)
Supplement: Supplementary file 13 — Additional file 13. Results: Ancestral area reconstructions inferred using BioGeoBEARS given the best tree under the best fitting model given A: DEC + J; B: DEC; and without range or dispersal constraint: C: DEC + J; D: DEC. For each model the single most probable state is shown first (boxes with areas at nodes) followed by the relative probability of each state represented with pie charts at nodes. Areas are represented by colours: Dark blue for Europe (E); green for Tropical Africa (T); yellow for Madagascar (M); light blue for Drakensberg (D); red for Cape (C); and further colours for widespread distributions as indicated in the legends. [file 12862_2019_1545_MOESM13_ESM.pdf]

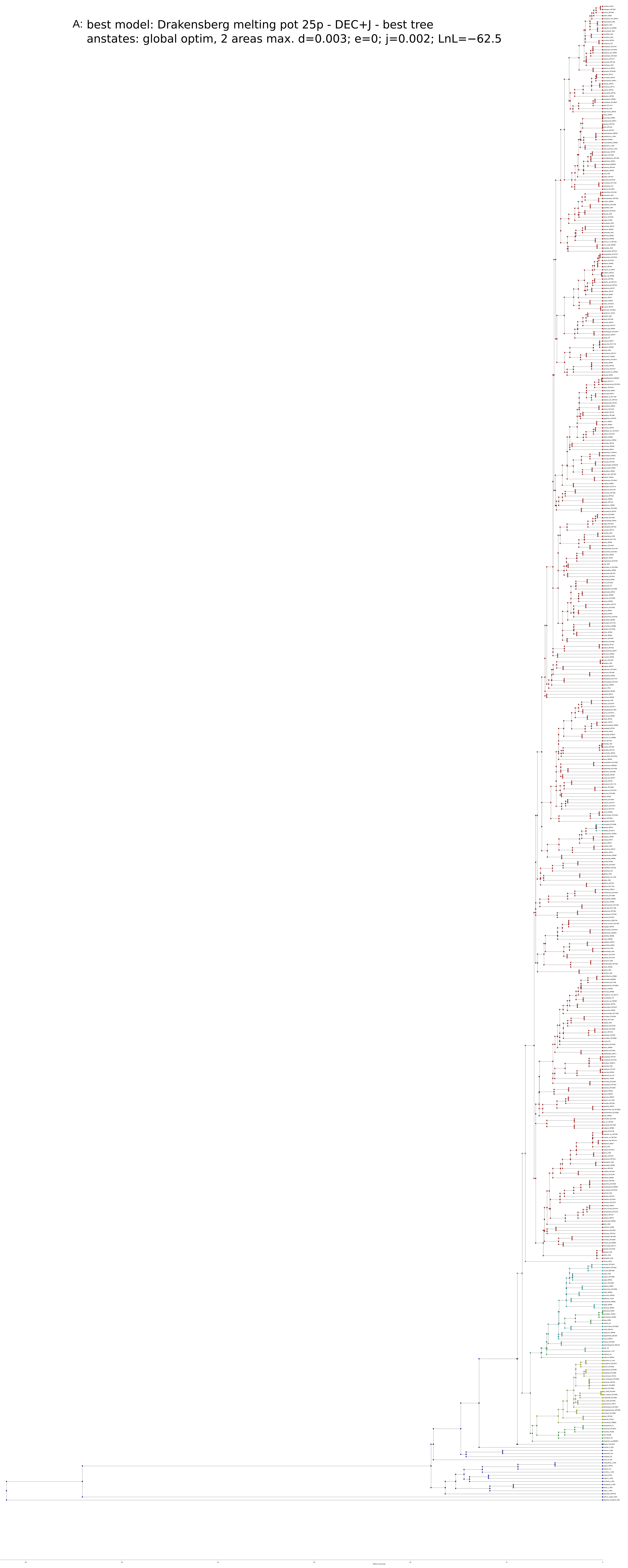
A: best model: Drakensberg melting pot 25p - DEC+J - best tree
anstates: global optim, 2 areas max. d=0.003; e=0; j=0.002; LnL=−62.5

A (2): best model: Drakensberg melting pot 25p - DEC+J - best tree
anstates: global optim, 2 areas max. d=0.003; e=0; j=0.002; LnL=−62.5

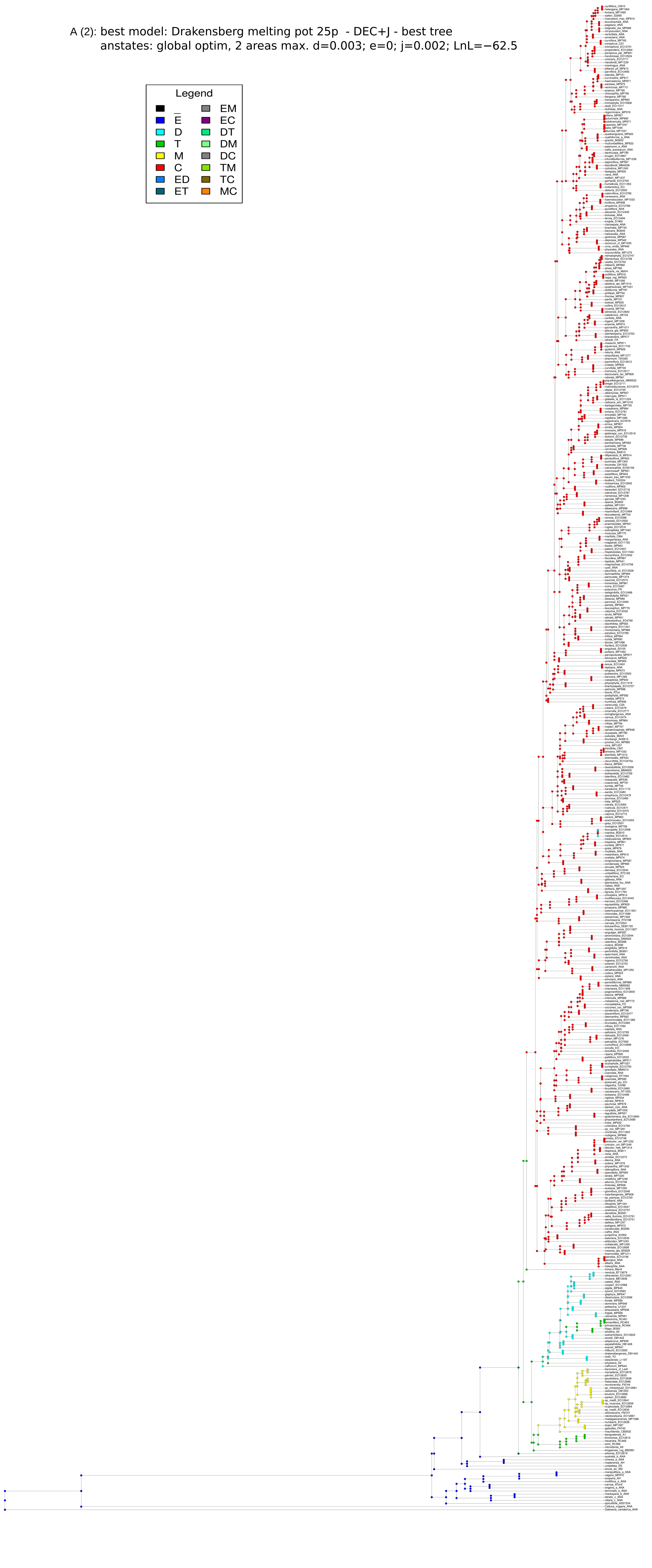

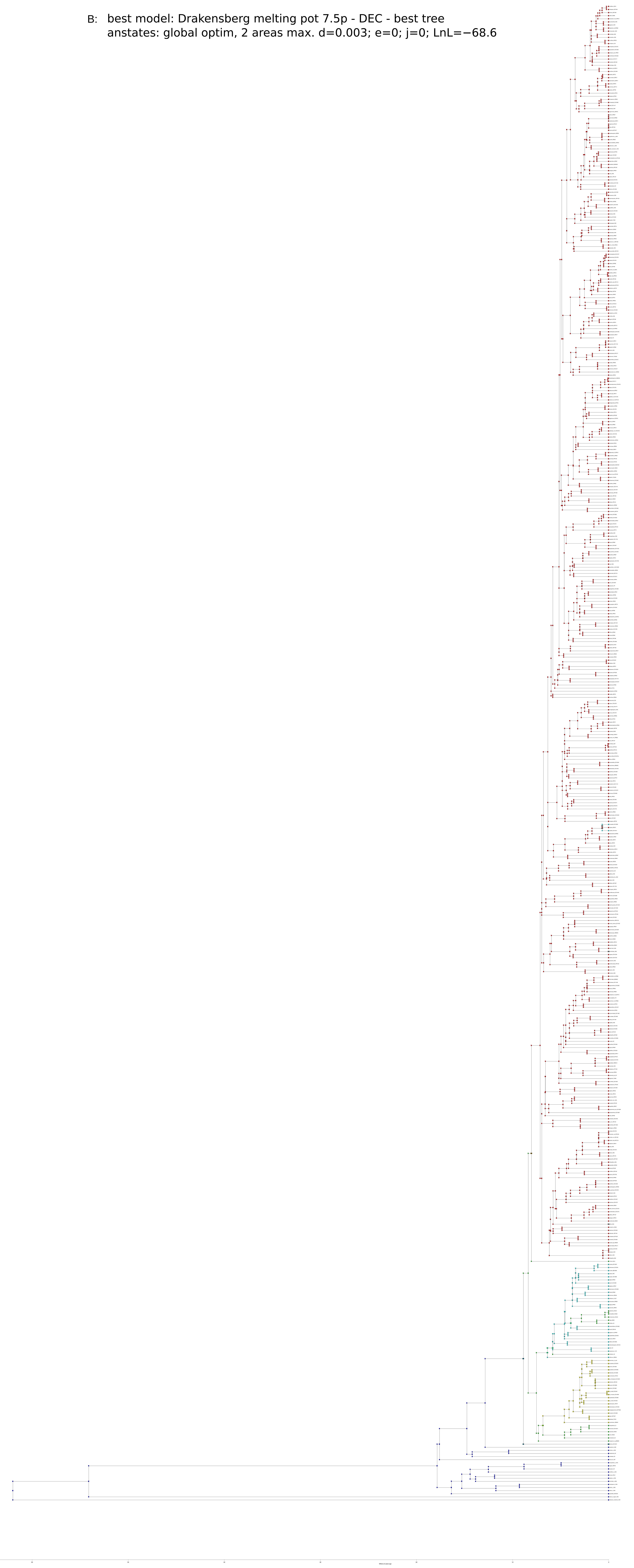
B: best model: Drakensberg melting pot 7.5p - DEC - best tree
anstates: global optim, 2 areas max. d=0.003; e=0; j=0; LnL=−68.6

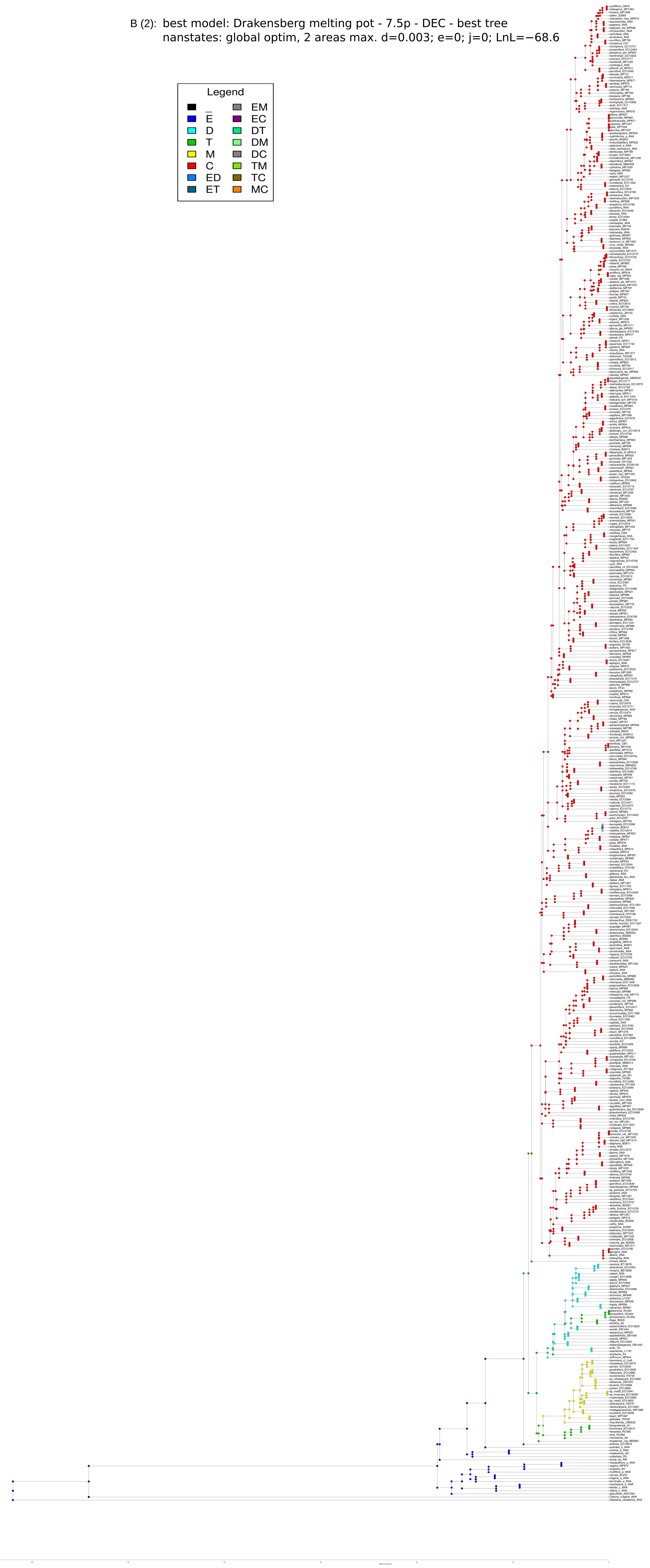
B (2): best model: Drakensberg melting pot - 7.5p - DEC - best tree
nanstates: global optim, 2 areas max. d=0.003; e=0; j=0; LnL=−68.6
Legend
_
E
D
T
M
C
ED
ET
EM
EC
DT
DM
DC
TM
TC
MC

C: noconstrain - DEC+J - best tree
anstates: global optim, 5 areas max. d=0.001; e=0; j=0.001; LnL=−70

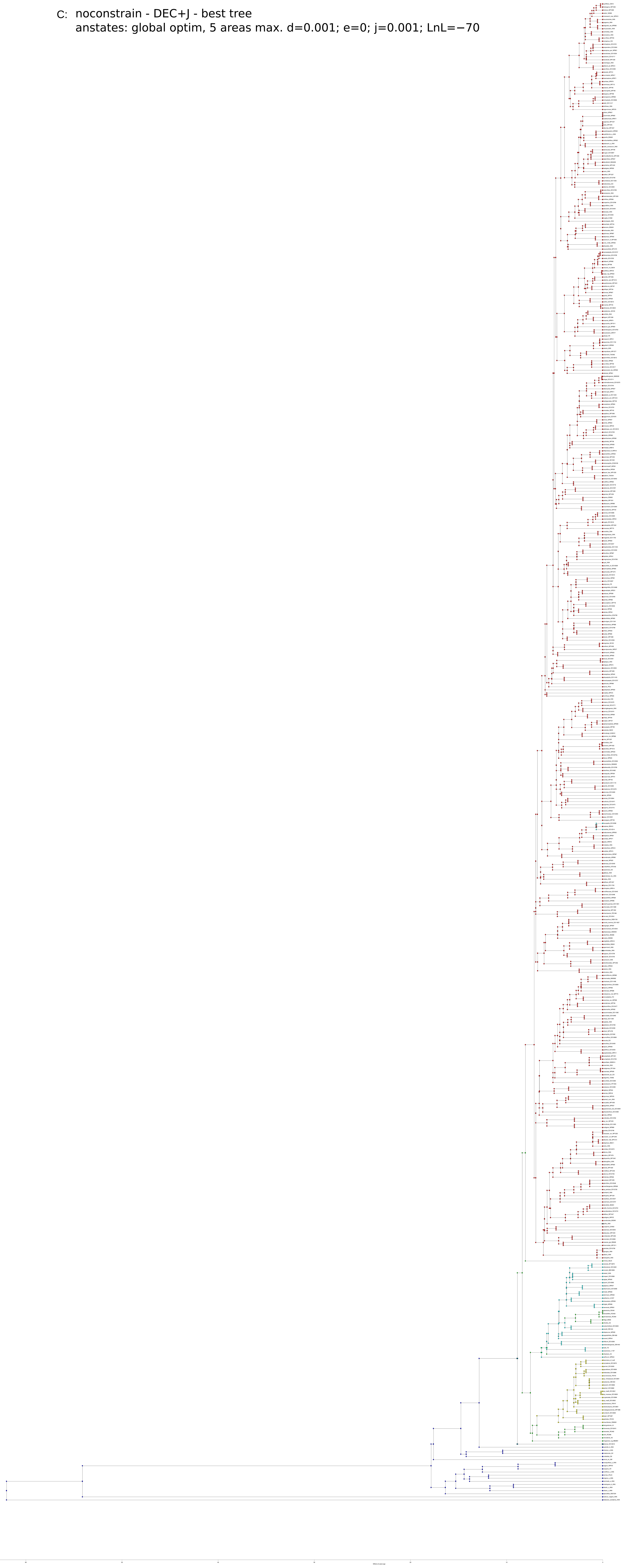

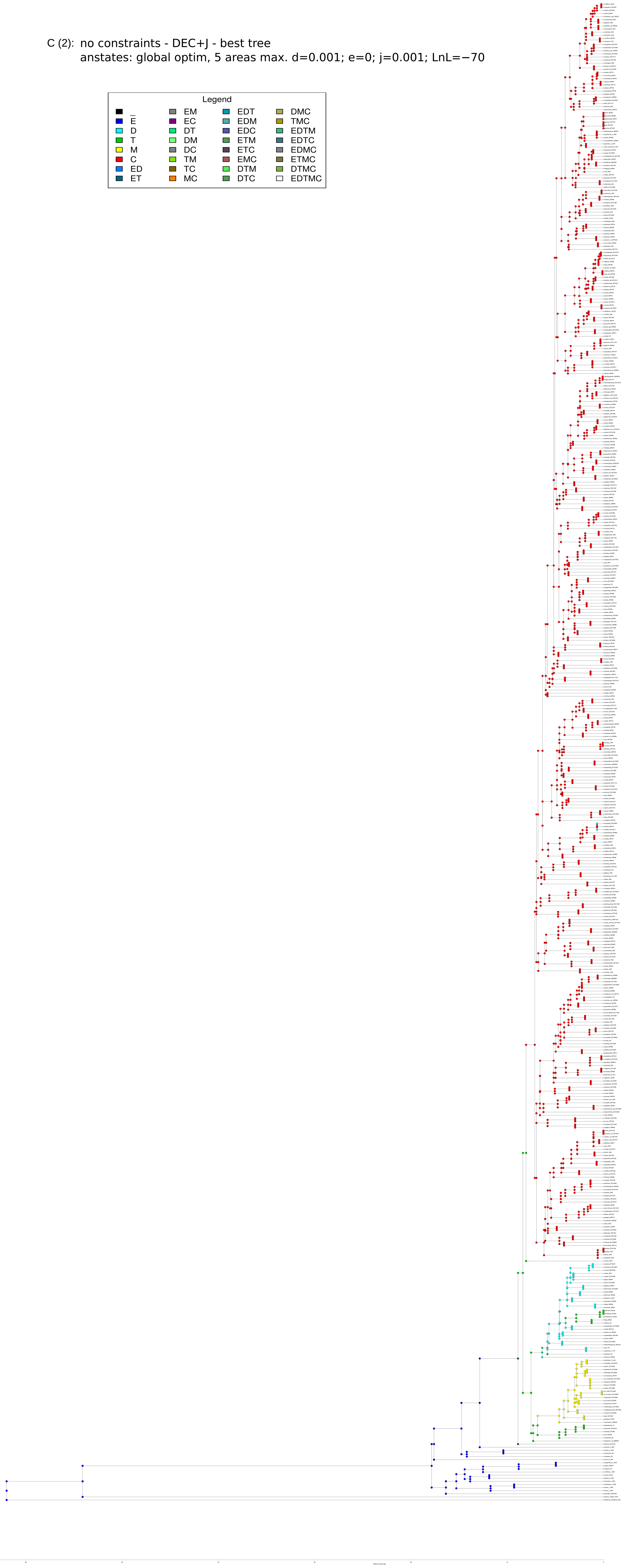

C (2): no constraints - DEC+J - best tree
anstates: global optim, 5 areas max. d=0.001; e=0; j=0.001; LnL=−70
Legend
_
E
D
T
M
C
ED
ET
EM
EC
DT
DM
DC
TM
TC
MC
EDT
EDM
EDC
ETM
ETC
EMC
DTM
DTC
DMC
TMC
EDTM
EDTC
EDMC
ETMC
DTMC
EDTMC

D: noconstrain - DEC - best tree
anstates: global optim, 5 areas max. d=0.001; e=0; j=0; LnL=−73.6

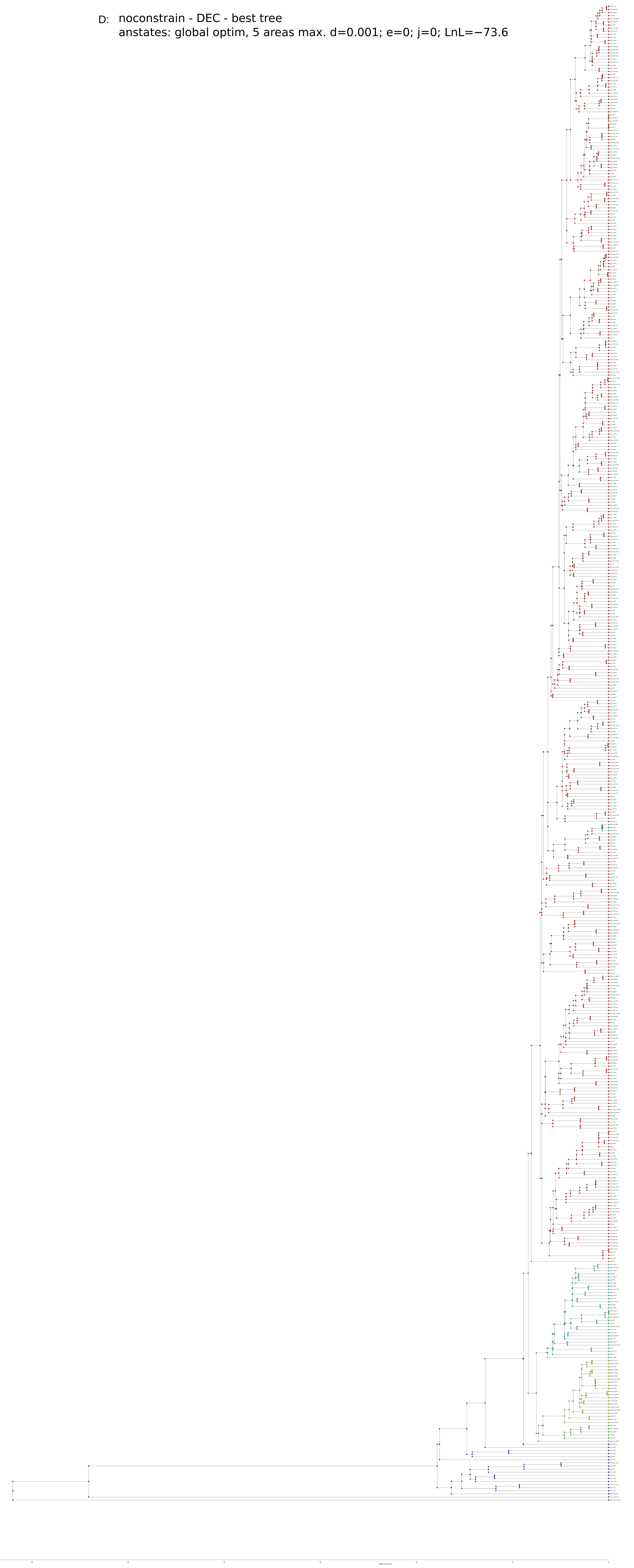

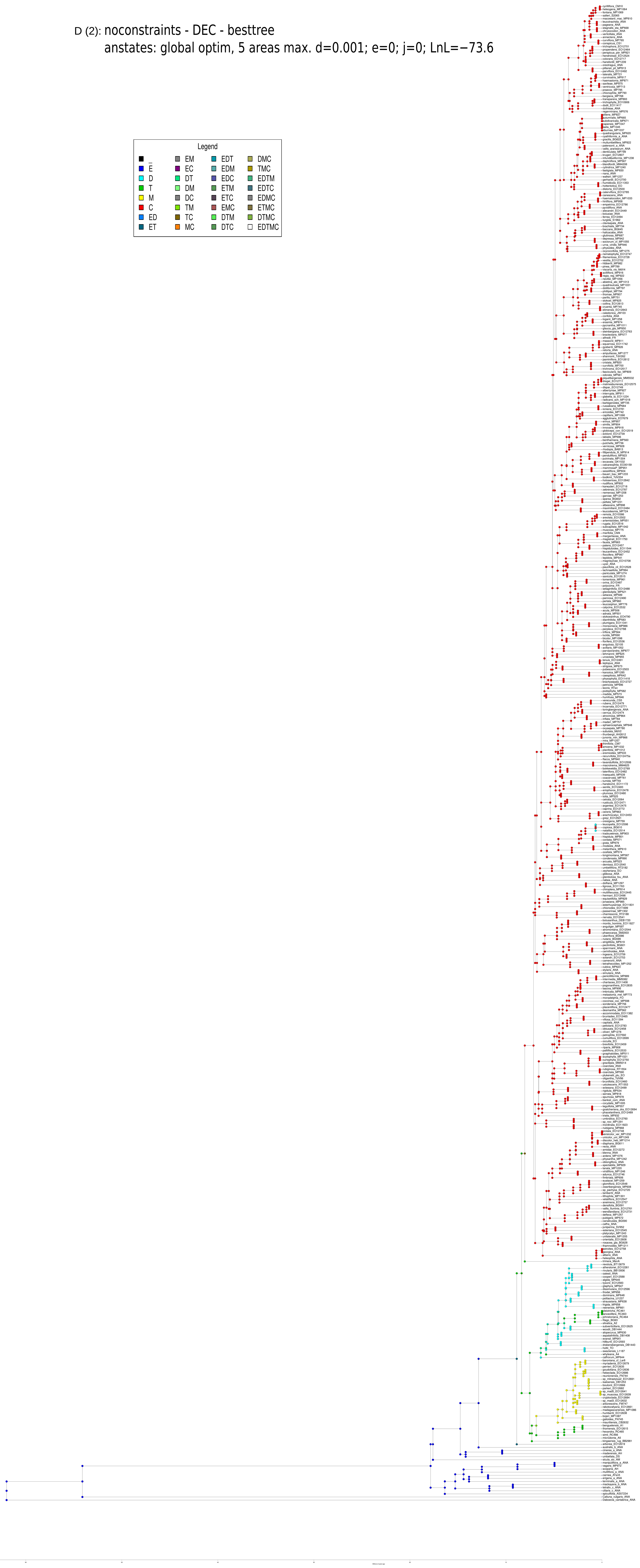
D (2): noconstraints - DEC - besttree
anstates: global optim, 5 areas max. d=0.001; e=0; j=0; LnL=−73.6
Legend
_
E
D
T
M
C
ED
ET
EM
EC
DT
DM
DC
TM
TC
MC
EDT
EDM
EDC
ETM
ETC
EMC
DTM
DTC
DMC
TMC
EDTM
EDTC
EDMC
ETMC
DTMC
EDTMC
